# Nanobody-based analysis of RhoGTPase stress response in *Schizosaccharomyces pombe*: Role of *mtl*2

**DOI:** 10.1016/j.btre.2026.e00963

**Published:** 2026-05-26

**Authors:** Noureen Rasheed, XiZe Ali, Tariq Ali, Faiz Muhammad, Muhammad Ali Rasheed

**Affiliations:** a Department of Biochemistry, University of Karachi, Karachi, Pakistan; b Department of Microbiology, University of Karachi, Karachi, Pakistan; c Department of Pharmaceutics, Faculty of Pharmaceutical Sciences, DOW University of Health Sciences, 74200, Karachi, Pakistan; d Department of Microbiology, Faculty of Life Sciences and Informatics, Balochistan University of Information Technology, Engineering and Management Sciences BUITEMS, 87300, Quetta, Balochistan, Pakistan

**Keywords:** Nanobodies, *mlt*2, Actin cytoskeleton, Fission yeast, V_H_H (P-36)

## Abstract

*Schizosaccharomyces pombe* (*S.pombe*), commonly referred to as fission yeast, is a widely utilised model organism for studying cellular responses to stress. The stress response mechanisms are largely conserved, offering insights into similar processes in higher eukaryotes, including humans.We have developed an in vivo nanobody-library screening platform in Δ*mtl*2 fission yeast subjected to lethal caspofungin stress, enabling the direct selection of nanobodies that improve survival. Through this method, we isolate a nanobody (Clone-C) that binds to and regulates the Rho1 GEF (Guanine nucleotide Exchange factor) Rgf1, enhancing cell survival and uncovering a hitherto unreported Mtl2–Rgf1–Rho1GTPase regulatory axis in the cell-wall stress response. This nanobody-based fluorescence-readout method offers a versatile tool for gene identification and antifungal target/ADC screening under certain stress conditions.

## Highlights

• An in vivo stress based nanobody screening platform in *S.pombe* Δ*mtl*2 under lethal caspofungin conditions.
• Identfification of nanobody clone C that improves survival and modulates *rgf*1/Rgf1 in Δ*mtl*2.
• Docking showing clone C binds Rgf1 outside its active Rho1 interacting sites.

## Graphical abstract

**Figure:**
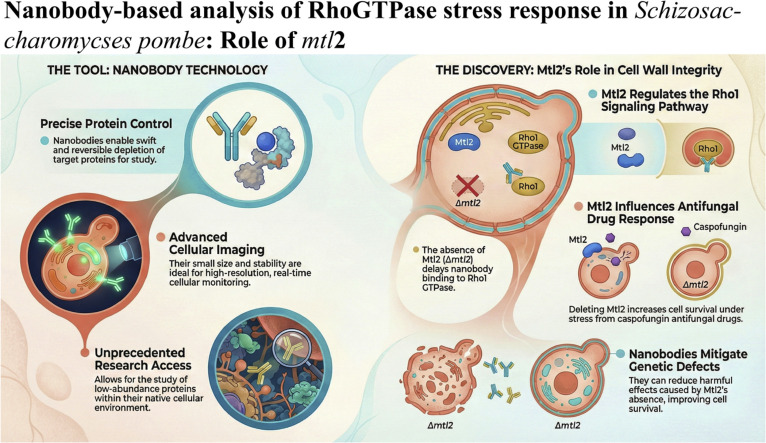

## 1 Introduction

**Fig. 1:**
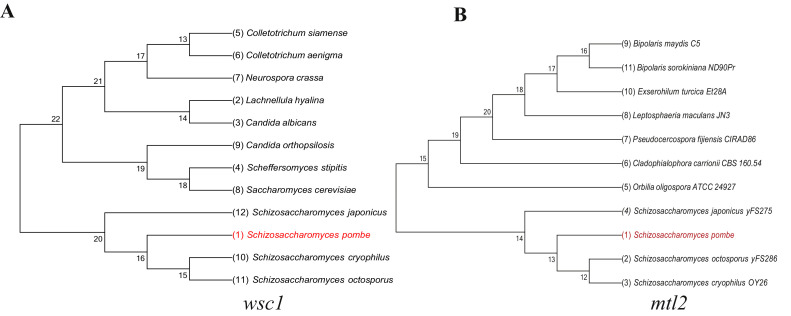
**Phylogenetic tree** illustrating the positioning of **A**. *wsc*1 and **B**. *mtl*2 within *S. pombe*. The bootstrap value based on 1000 replications is indicated as the branching point with MEGA-7.

**Fig. 2:**
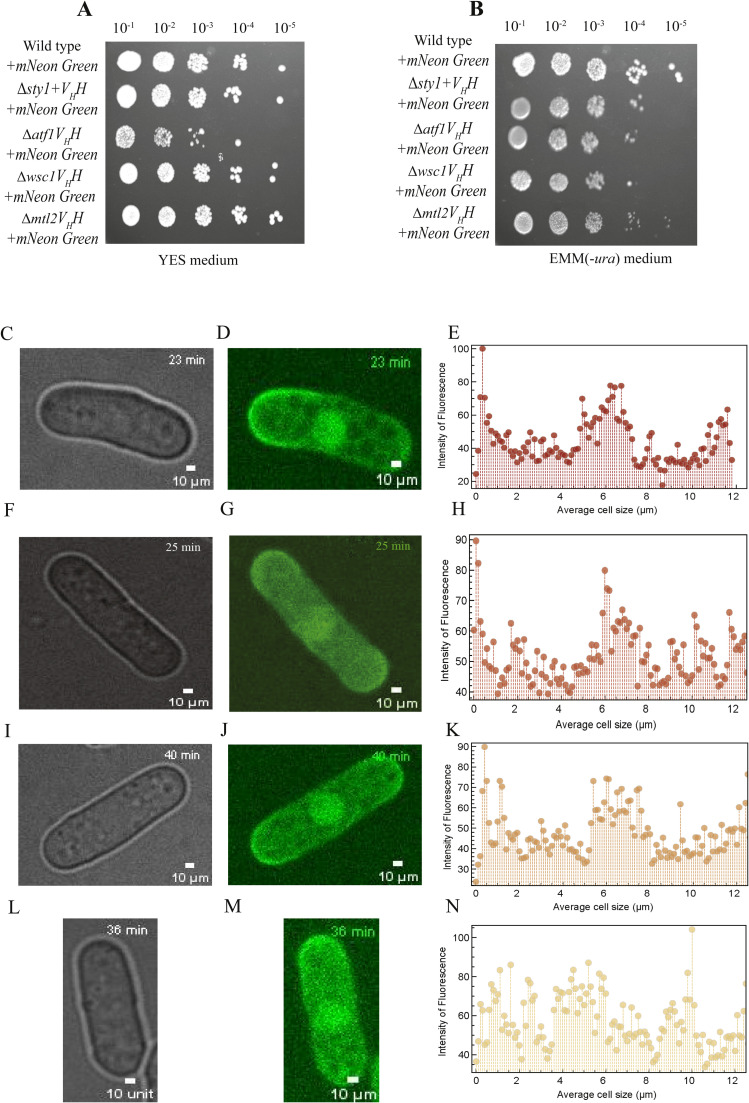
**P**‑**36 nanobody reports delayed terminal localization in Δ*mtl*2 compared with wild type, indicating altered Rho1**‑**pathway activation dynamics under non‑stress conditions. Spot testing A**. YES medium and **B**. EMM supplemented with all components except uracil (-ura) medium of *S. pombe* with P-36 nanobodies and with deletion mutant of Δ*sty*1, Δ*atf*1, Δ*wsc*1 and Δ*mtl*2 genes. **C**. DIC imaging of wild type (AL07), **D**. Confocal GFP imaging with P-36 in wild type (AL07), **E**. Graph depicting fluorescence intensity in YES medium. **F**. DIC of Wild type (AL07), G. Confocal GFP with P-36 in wild type (AL07), **H**. Graph of fluorescence intensity in EMM supplemented with all components except uracil (-ura) medium. **I**. DIC of Δ*mtl*2 (AL16), **J**. Confocal GFP with P-36 in Δ*mtl*2 (AL16), **K**. Graphical Illustration depicting fluorescence intensity in YES medium. **L**. DIC of Δ*mtl*2 (AL16), **M**. Confocal GFP with P-36 in Δ*mtl*2 (AL16), **N**. Graphical representation of fluorescence intensity in EMM supplemented with all components except uracil (-ura) medium.

Fungal and yeast cells possess a strong cell wall that must endure continuous mechanical and environmental stressors. This wall contains surface sensor proteins that monitor alterations in cell wall integrity and transmit this information into the cell to initiate adaptive responses [1]. This wall contains surface sensor proteins that monitor alterations in cell wall integrity and transmit this information into the cell to initiate adaptive responses. (Fig. 1 A and B and 2, Phylogenetic tree) [2,3]. This activation subsequently regulates cell wall repair through protein kinase C and the Pmk1 MAP kinase cascade in response to various stresses, including heat and antifungal agents. Both sensors have analogous domain architecture, featuring a cytoplasmic C-terminal tail for downstream signaling and a single transmembrane segment [4,5]. Both sensor types exhibit a comparable design, characterized by a cytoplasmic C-terminal tail that enables downstream signaling, followed by a singular transmembrane domain.

Alongside the cell wall integrity route, *S. pombe* initiates an extensive stress-activated protein kinase (SAPK) response facilitated by the Sty1–Atf1 pathway. The activation of Sty1 results in its phosphorylation and subsequent nuclear translocation, where it activates the transcription factor Atf1 to trigger genes associated with stress adaptation, DNA repair, and cell-cycle regulation, thus facilitating survival under unfavourable conditions [6,7]. Research underscores the importance of the Sty1-Atf1 pathway in orchestrating stress responses, especially in regulating gene expression during the construction of fungal reproductive structures and in response to rapamycin treatment [8,9]. While Mtl2 and Wsc1 are recognized as activators of Rho1 and the Pmk1 pathway, their specific functions in regulating the Sty1–Atf1 cascade are inadequately elucidated, highlighting a notable deficiency in the comprehension of how cell wall sensors interact with overarching stress signalling networks [10]. Clarifying these connections is essential not just for fundamental yeast biology but also for comprehending similar processes in human fungal infections and for formulating methods to address antifungal resistance.

Yeast stress responses include both specialized adaptations to particular stressors and a conserved environmental stress response that engages overlapping groups of genes and transcription factors. Cells subjected to mild stress can develop tolerance to more intense doses of the same or even disparate stressors, suggesting the presence of universal adaptation mechanisms [11,[12], [13], [14]]. In budding yeast, conserved environmental stress response genes and transcription factors like Msn2/4 orchestrate the activation of stress-protective functions, while similar regulatory networks involving Rho1GTPase, Sty1 MAPK, and Atf1 underscore the intricacy of stress adaptation in fission yeast [[15], [16], [17],^13^,^18^,^19^]. Due to its genetic manipulability and conserved signaling pathways, *S. pombe* serves as an effective model for elucidating cellular mechanisms of environmental and drug-induced stress responses Rho1GTPas is a pivotal element in the cell wall integrity network, with its activation and location meticulously regulated by guanine nucleotide exchange factors like Rgf1. Rgf1 is essential for the location of actin patches at the cell apexes, hence facilitating cell polarity, morphogenesis, and septation. The Mtl2 sensor is regarded as a crucial upstream activator of Rho1/Pck1 signalling and is vital for preserving cell wall integrity; the absence of Mtl2 disrupts glucan synthase activity, diminishes β-glucan levels, and induces hypersensitivity to antifungals targeting the cell wall, which can be partially mitigated by moderate overexpression of Rho1GTPase. The molecular processes connecting Mtl2 to specific Rho1GTPase regulators, such as Rgf1, under particular stress situations, like caspofungin exposure, remain inadequately elucidated.

Simultaneously, there is an increasing interest in employing nanobodies single-domain antibody fragments originating from camelid heavy-chain antibodies as exact instruments to investigate and modulate protein function in vivo. Traditional antibody production typically necessitates animal vaccination and labor-intensive phage display choices, while antibodies derived from non-immune libraries may exhibit comparatively low affinity [12,[20], [21], [22]]. In contrast, nanobodies are diminutive, stable, readily produced, and can be conjugated to fluorescent reporters without substantially compromising binding, rendering them appealing for live-cell imaging and functional perturbation [23,24]. Recent advancements in synthetic V_H_H library development and in vivo selection methodologies now facilitate the fast generation and screening of nanobodies without the need for prolonged animal immunization.

This study expands upon a previously established pDUAL-based nanobody screening approach in *S. pombe*, with the objective of connecting nanobody-mediated protein targeting to stress-response genetics within the framework of Mtl2-dependent signaling. This study specifically examines RhoGTPase-specific nanobodies under various stress circumstances, including osmotic and oxidative stress, as well as exposure to the cell wall-active antifungal caspofungin, concentrating on the *mtl*2 sensor [25,26]. The study aims to identify nanobodies and their targets that promote survival by expressing a synthetic V_H_H library fused to the mNeonGreen reporter in Δ*mtl*2 cells under lethal caspofungin stress and selecting for surviving fluorescent clones. Additionally, it seeks to ascertain whether these nanobodies can uncover previously unrecognized regulatory connections among Mtl2, Rgf1, Rho1GTPase, and downstream stress-response pathways [27,28]. This nanobody-based gene identification technology not only enhances the comprehension of fungal stress adaptation but also holds potential for antifungal target discovery and antibody-drug conjugate (ADC) screening under specific stress situations.

## 2 Materials and methods

### 2.1 Construction of Δ*sty1*, Δ*atf1*, Δ*wsc1*, and Δ*mtl2* mutants

The following constructs were created by amplifying the 5′ UTR and 3′ UTR from the wild-type genomes of *sty1, atf1, mtl*2 and *wsc*1, substituting the ORF region with a hygromycin resistance gene. Following the cultivation of the wild type culture to an OD_600_ of 0.5–0.8, 1 µg of the linearized fragment from the pertinent plasmid comprising the 5′UTR-hygromycin-3′UTR region is incorporated into pBlueScript II SK. The ligation was performed by Gibson assembly. The plasmid was transformed into *E.coli* to generate multiple copies. The isolated plasmid containing the hygromycin resistance gene was linearly amplified by PCR. The overnight culture of fission yeast in YES medium, with an OD_600_ adjusted between 1.2 and 1.5, was centrifuged at 3000 rpm for 5 min. Eliminate the supernatant and resuspend the cell pellet in 1x TE 0.1 M LiOAc. Wash it with 1x TE 0.1 M LiOAc three times in a 1.5 mL Eppendorf tube and incubate at 32 °C for one hour. Incorporate 5 µL of hygromycin with the corresponding 5′ UTR - 3′ UTR DNA. Subject the cells to thermal shock for 5 min at 42 °C. Centrifuge the cells for 2 min at 3000 rpm [29]. Resuspend the cell pellet in 200 µL of sterile deionized water. Inoculate the cells into YES medium plates augmented with 50 mg/mL hygromycin. Plates were incubated for four to five days at 30 °C; isolated clones were selected and cultured in YES broth augmented with hygromycin. Following genomic extraction, a PCR reaction was conducted to verify the presence of the mutation. **Supplementary Figures** S1, S2, S3, and S4; Table 1, Table 2. The sequence validation was conducted by submitting the material for sequencing to Sangon Biotech (Shanghai) Co., Ltd

**Table 1:** delineates the organisms and strains employed.

Strains code	Genotype	Source
AL01	h^-^ 972	[30]
HM123	h^-^ leu1–32	[31]
PN513	h^-^ leu1–32 ura4-D18	Nurse Lab stock
AL02	h^-^*sty1*::hphMX6^R^ leu1–32 ura4-D18	This study
AL03	h^-^*atf1*::hphMX6 ^R^ leu1–32 ura4-D18	This study
AL04	h^-^*wsc1*::hphMX6 ^R^ leu1–32 ura4-D18	This study
AL05	h^-^*mtl2*::hphMX6 ^R^ leu1–32 ura4-D18	This study
AL06	h^-^ leu1–32 ura4-D18:pDUAL+mNeonGreen:ura4	[25]
AL07	h^-^ leu1–32 ura4-D18:pDUAL+V_H_H(P-36)+mNeonGreen:ura4	[25]
AL08	h^-^*sty1*::hphMX6 ^R^:pDUAL+mNeonGreen:ura4	This study
AL10	h^-^*sty1*::hphMX6 ^R^:pDUAL V_H_H(P-36)+mNeonGreen:ura4	This study
AL11	h^-^*atf1*::hphMX6 ^R^:pDUAL+mNeonGreen:ura4	This study
AL12	h^-^*atf1*::hphMX6 ^R^:pDUAL V_H_H(P-36)+mNeonGreen:ura4	This study
AL13	h^-^*wsc1*::hphMX6 ^R^:pDUAL+mNeonGreen:ura4	This study
AL14	h^-^*wsc1*::hphMX6 ^R^:pDUAL V_H_H(P-36)+mNeonGreen:ura4	This study
AL15	h^-^*mtl2*::hphMX6:pDUAL+mNeonGreen:ura4	This study
AL16	h^-^*mtl2*::hphMX6 ^R^:pDUAL V_H_H(P-36)+mNeonGreen:ura4	This study
AL17	h^-^*mtl2*::hphMX6 ^R^: *rgf*1::kanMX6 ^R^	This study
AL18	h^-^*mtl2*::hphMX6 ^R^: *rgf*1::his6^+^	This study
AL19	h^-^*mtl2*::hphMX6 ^R^: *rgf*1::his6^+^pDUAL+mNeonGreen:ura4	This study
AL20	h^-^*mtl2*::hphMX6 ^R^: *rgf*1:: his6^+^pDUAL V_H_H(P-36)+mNeonGreen:ura4	This study

**Table 2:** Catalog of plasmids utilised.

Description	Reference or source
pBlueScript II SK+ vector (Amp+)	**Purchased from Add gene catalogue # 212,205**
pDUAL-Pef1a (Amp+)	**DNA bank Catalog # RDB06529**
pBluescript II SK+5′UTR+*sty1* (HYG)+3′UTR	Constructed in this Study
pBluescript II SK+5′UTR+*atf1* (HYG)+3′UTR	Constructed in this Study
pBluescript II SK+5′UTR+*wsc1* (HYG)+3′UTR	Constructed in this Study
pBluescript II SK+5′UTR+ *mtl2* (HYG)+3′UTR	Constructed in this Study
pDUAL+linker+mNeon	[25]
pDUAL-V_H_H(P-36)+linker+mNeon	[25]
pEX-N-His-GST	**Cat# PS100028**
pBluescript II SK 5′UTR+*rgf1* (Kena)+3′UTR	Constructed in this Study

### 2.2 Plasmid and cloning techniques

Plasmids were constructed by digesting the pDUAL-Pef1a vector plasmid with *NheI* and *Bam*HI enzymes. This plasmid encodes a protein derived from cloned genes and enables cellular entrance through chromosomal integration and the incorporation of multicopy DNA. Digesting the multicopy plasmid with a particular restriction endonuclease prior to transformation into yeast cells facilitates the production of the necessary chromosomal integration segment. The designated V_H_H (Variable Heavy domain of Heavy Chain) region, encompassing CDRx9 (complementarity-determining region), was conjugated utilizing a specified sequence (5′-GGTGCCGGCGCAGGAGCG-3′), followed by the incorporation of the mNeonGreen reporter gene at the C-terminus. A *Sal1* endonuclease site was deliberately positioned between the V_H_H and the linker to facilitate the creation of a varied V_H_H library. The reporter gene was concluded with an Ochre stop codon. It has a likelihood of 0.59, indicating it is a favored stop codon for the S. *pombe* expression. Sequences are included in **Supplementary Table** S1

### 2.3 Designing and constructing libraries

The inferior quality of non-immune libraries may originate from various factors. The antibody scaffold may improperly fold and display the complementarity-determining region. The inadequate management of CDR region diversity may persist, perhaps leading to a rise in erroneous clones due to unforeseen mutations or empty clones. Researchers developed synthetic diversity in the three complementarity-determining regions (CDRs) by systematically altering each location of CDR1 and CDR2 with a specific array of amino acids that partially mimics natural variation. This approach significantly diminishes highly hydrophobic residues to mitigate aggregation. Gene Script was employed to generate a varied synthetic V_H_H library, wherein CDR1 and CDR2 uniformly comprised 7 amino acids, whereas the lengths of Camelidae V_H_H CDR3 exhibit intrinsic variety, a loop recognized for its influence on antigen binding selectivity. A comprehensive assessment of natural V_H_H diversity was employed to create a synthetic design intended to reduce hydrophobicity in targeted regions. Until 1.5 × 10^9, the potential for diversification remained consistent. GeneScript, located in Piscataway, NJ 08854, USA, has created the pDUAL-Pef1a library, with random recombination sequences for CDR1, CDR2, and CDR3 [25].

### 2.4 Selection of transformants of positive clones

The V_H_H library plasmid was converted utilizing chemical agents (lithium acetate and polyethylene glycol) and thermal stresses, facilitating the transfer of genetic material across the cell wall and plasma membrane with minimal disruption to the transferred DNA or recipient cells. The effectiveness ranged from 1.0 × 10^3 to 1.0 × 10^4 transformants per microgram of plasmid containing 10^8 cells of *S. Pombe* cells [29]. Subsequent to the transformation, the relevant V_H_H library, at a concentration of 1 µg/µL in 20 mL of yeast grown in YES medium with an optical density of 0.3–0.6 OD_600_, was transferred to Edinburgh Minimal Medium (EMM) (Coolaber, CAT # PM4531), supplemented with all components except uracil (-ura) (Table 3), for a duration of 5 days to isolate positive clones. Elucidates the composition of the medium. Each clone was extracted from the plate and assessed for fluorescence. Fluorescent clones exhibiting positivity were lysed using a genomic isolation technique (Tianamp yeast DNA kit, CAT # DP307–02) (**Supplementary Table S2**). The segment was amplified using the Forward primer 5′-ctgtttcatcgaactcacgctagcATGGAAGTCCAGCTGCAGGCGTCCG -3′ and the Reverse primer 5′-atttagaagtggcgcgccggatccTTActtgtacagctcgtccatgccc-3′, and subsequently integrated into the pDUAL-Pef1a plasmid [32] (**Supplementary Table S3**), which had been digested with *Nhe*1 and *BamH*1 endonucleases. The vector was modified in *Escherichia coli* strain (D-105β) grown in LB medium with ampicillin selection. The plasmid was isolated with the Tianprep micro plasmid kit (Cat # DP103–03) and subsequently digested with 1 µg/µL of *Not*1 enzyme. The linearized pDUAL-Pef1a plasmid was introduced into fission yeast and cultivated in EMM (Coolaber, CAT # PM4531) [33], with all supplements provided except for -uracil. Clones were isolated and thoroughly analyzed.

**Table 3:** Composition of Utilised Media.

Yes Medium (with all supplements)		EMM Medium (with all supplements)	
Ingredients	Weight	Ingredients	Weight
Yeast Extract	= 5 g	EMM	= 31.24 g
Glucose	= 30 g	Adenine	= 0.225 g
Adenine	= 0.225 g	Histidine	= 0.225 g
Histidine	= 0.225 g	Leucine	= 0.225 g
Leucine	= 0.225 g	Lysin	= 0.225 g
Uracil	= 0.225 g	Note:	Add Ca Mg After sterilization
Lysine	= 0.225 g	Ca Mg (liquid)	= 10mL
AGAR	= 20 g	AGAR	= 20 g

### 2.5 Spot testing

#### 2.5.1 Effect of sorbitol, potassium chloride (KCl), and hydrogen peroxide (H_2_O_2_)

Yeast cells were cultivated overnight to the mid-logarithmic exponential phase (OD_600_ = 0.4–0.6) in YES broth (with all supplements) and EMM supplemented with all components except uracil (-ura) while agitated at 220 rpm. The growth was subjected to varying concentrations of 0.5 M and 0.6 M KCl at 30 °C in plates. Sorbitol concentrations of 1.5 M and 2.0 M in the plate. Concentrations of H_2_O_2_ utilized were 10 mM and 15 mM in the plates. The growth was diminished tenfold and subsequently monitored on the suitable media. 2 mL of culture were utilized for microscopy following centrifugation at 500xG for five minutes, after which the supernatant was discarded. Resuspended in the fresh medium accordingly. The OD_600_ was adjusted to 0.1, followed by the application of 0.15 µL onto the plates subsequent to serial dilution. Plates were incubated for four days at 30 °C. The identical concentration of corresponding stress compounds was utilized in broth (YES medium with all supplements) and EMM supplemented with all components except uracil (-ura) at 30 °C. The culture was centrifuged at 500xG for five minutes and resuspended in 2 mL, from which 0.15 µL of the pellet was applied to a slide for microscopic imaging.

#### 2.5.2 Impact of caspofungin (Csp)

The effect of caspofungin (Cat10011218, Sinopharm Chemicals) on Δ*wsc1* and Δ*mtl2* was assessed using spot testing. Mutant strains were incubated overnight to attain an optical density (OD_600_) of 0.5–0.8. Subsequently, both the wild-type strain and the mutants were inoculated into caspofungin-containing plates with concentrations of 0.5 and 2.0 µg/mL of caspofungin in YES medium, followed by a 4-day incubation at 30 °C. The identical protocol was employed for mutants containing the vacant pDUAL-Pef1a plasmid labelled with the mNeonGreen gene; however, they were evaluated on EMM supplemented with all components except uracil (-ura) plates with concentrations of 0.5, 1.0, 1.5, and 2.0 µg/mL of caspofungin, and incubated for four days at 30 °C.

### 2.6 Isolation of nanobodies utilising the pDUAL-Pef1a plasmid

The organism was cultured overnight at 30 °C in EMM supplemented with all components except uracil (-ura) broth. Inoculate fresh EMM supplemented with all components except uracil (-ura) media with the overnight culture and set the optical density at 600 nm to 0.3–0.4. After two hours of growth, commence the lithium oxide transformation process with 1 µg of randomly generated pDUAL-Pef1a library plasmids containing mNeonGreen-tagged nanobodies from Gene Script in Δ*mtl2*, grown with a lethal dose of caspofungin at 1.8 µg/µL. The medium was incubated at 30 °C for four days. Ten 90 mm plates were utilized using EMM supplemented with all components except uracil (-ura) medium for this purpose. The isolated colonies were stored at -80 °C in 1.5 mL Eppendorf tubes with 15% glycerol.

### 2.7 Analysis of growth curves

Each strain of fission yeast was cultured in YES (Yeast Extract Supplement) medium, while EMM (Edinburgh Minimal Media) supplemented with all components except uracil (-ura) was employed for the selection of the modified plasmid carrying V_H_H nanobodies. After overnight proliferation, the organism concentration was adjusted to 0.1 at OD_600_ and then transferred to 200 µL in a Costar 96-well flat-bottom plate. The growth curve investigation was performed utilizing an Infinite 200 Pro instrument, serial number 1811012833.

### 2.8 Quantitative polymerase chain reaction (qPCR) analysis

Among all the isolated clones, clone C was the most resistant and demonstrated fluorescence under the fluorescent microscope. Consequently, clone-C was cultured in EMM supplemented with all components except uracil (-ura) medium for 8 hours at 30 °C with the incorporation of hygromycin. Total RNA was extracted utilizing an RNA extraction kit, and qPCR analysis was conducted with the designated primers for the gene expression of *rgf1, rgf3, gef1*, and *pmk1* (Supplementary Table S4).

### 2.9 Protein purification and analysis

*S. pombe* (AL-18 and AL-20) strains containing 6His (5 mL) at an OD_600_ of 0.5, in the presence of 1.8 μg of Caspofungin, were pelleted immediately upon the addition of 10% TCA and subsequently washed in 20% TCA. The pellets were resuspended in 100 μl of 12.5% TCA, supplemented with glass beads, and lysed by vortexing for 5 min. Cell lysates were centrifuged, washed with cold acetone, and dried at 55 °C for 15 min. Pellets were resuspended in 50 μl of a solution comprising 1% SDS, 25 mM Tris–HCl (pH 8.0), and 1 mM EDTA. Samples were subjected to electrophoretic separation using SDS-PAGE (4 -15% MiniProtein Gel, BioRad) and subsequently analyzed via Western blotting with anti-mouse antibodies (Bio-Rad, AB_11125547).

### 2.10 Investigation of rgf1 binding domain dynamics and structure

For protein-protein interaction analysis, download the Rgf1 protein structure (sequence code = AF-Q9Y7U6-F1) for *S. pombe* from the AlphaFold protein structure database at the following link: https://alphafold.ebi.ac.uk/entry/Q9Y7U6. The Rgf1 protein structure and V_H_H derived from clone-C were subjected to 3D protein structure prediction using SWISS-MODEL (https://swissmodel.expasy.org/) [34] by inputting the amino acid sequence. The prediction of potential active sites was conducted by inputting the PDB structures into SPPIDER (http://sppider.cchmc.org/) [35,36]. Upon obtaining the potential site, both the receptor/acceptor (3D structure of the Rgf1 protein) and the donor (V_H_H protein) were sent to HADDOCK (https://wenmr.science.uu.nl/haddock2.4/), where the interaction between Rgf1 and V_H_H was predicted.

### 2.11 Spinning disk confocal microscopy for in vivo cellular imaging

A Nikon Eclipse Ti2-E microscope, a Nikon 60X oil immersion objective lens (NA 1.40, Plan Apochromat Lambda), a spinning-disk system (Yokogawa Electric Corporation, CSU-W-1), a Photometrics Prime 95B sCMOS camera, a Piezo Z stage (Physik Instrumente), and a live cell stage were utilised. The images were obtained with Metamorph, featuring a z-step increase of 0.5 µm and an x-y plane resolution of 183.33 nm per pixel. Laser wavelengths of 488, 561, or 640 nm are utilised to stimulate the fluorophores. To evaluate the sensitivity of the WT and V_H_H (P-36), yeast cells were grown exponentially in YES medium overnight.

### 2.12 Statistical examination

Statistical analyses were conducted using Prism version 9.0 (GraphPad Software, LLC) and RStudio version 2021.09.1 Build 372. (**Supplementary Table S2)** lists the tools and software employed in the study.

## 3 Results

### 3.1 Transformation of pDUAL-Pef1a into recipient fission yeast cells

The prevalence of pDUAL-Pef1a with V_H_H in the transplanted fission yeast cells was significantly elevated on EMM supplemented with all components except uracil (-ura) plates, as illustrated in Supplementary Fig-5. Upon introducing 5 µL of plasmid from the synthetically constructed library into 50 mL of fission yeast recipient cells, we observed a substantial number of colonies on a 150 mm plate. This may significantly enhance nanobody screening efficiency.

### 3.2 Spot testing

Following the introduction of the P-36 nanobody into the deletion mutants Δ*sty*1, Δ*atf*1, Δ*wsc*1, and Δ*mtl*2, spot testing was performed in YES medium (Fig. 2A) and in EMM supplemented with all components except uracil (-ura) (Fig. 2B Spot Test). The wild type of fission yeast demonstrated terminal localisation with GFP at 23 min in YES medium (Fig. 2C, D, and E) (Supplementary Videos S1 and S2 at https://doi.org/10.6084/m9.figshare.28756475.v1) and at 45 min in EMM supplemented with all components except uracil (-ura) (Fig. 2F, G, and H) (Supplementary Video S3 and S4 at https://doi.org/10.6084/m9.figshare.28756475.v1). In the presence of P-36 nanobody, Δ*mtl*2 cells exhibited fluorescence approximately 40 min after being introduced to YES medium (Fig. 2I, 2J, and 2K) (Supplementary Video S5 and S6 at https://doi.org/10.6084/m9.figshare.28756475.v1). and in 36 min in EMM supplemented with all components except uracil (-ura) medium. Refer to Fig. 2L, M, and N, as well as Supplementary Videos S7 and S8 available at https://doi.org/10.6084/m9.figshare.28756475.v1). The delayed appearance of P-36 fluorescence at cell tips in Δ*mtl*2 relative to wild type suggest that loss of the Mtl2 sensor slows activation of the Rho1-dependent polarity machinery, even in the absence of exogenous stress. Because ura4⁺ pDUAL‑Pef1a plasmids are not stably maintained in YES without selection, we base all conclusions on experiments performed in EMM supplemented with all components except uracil (-ura); YES data are shown only as supportive, non‑quantitative controls.

#### 3.2.1 Impact of sorbitol

**Fig. 3:**
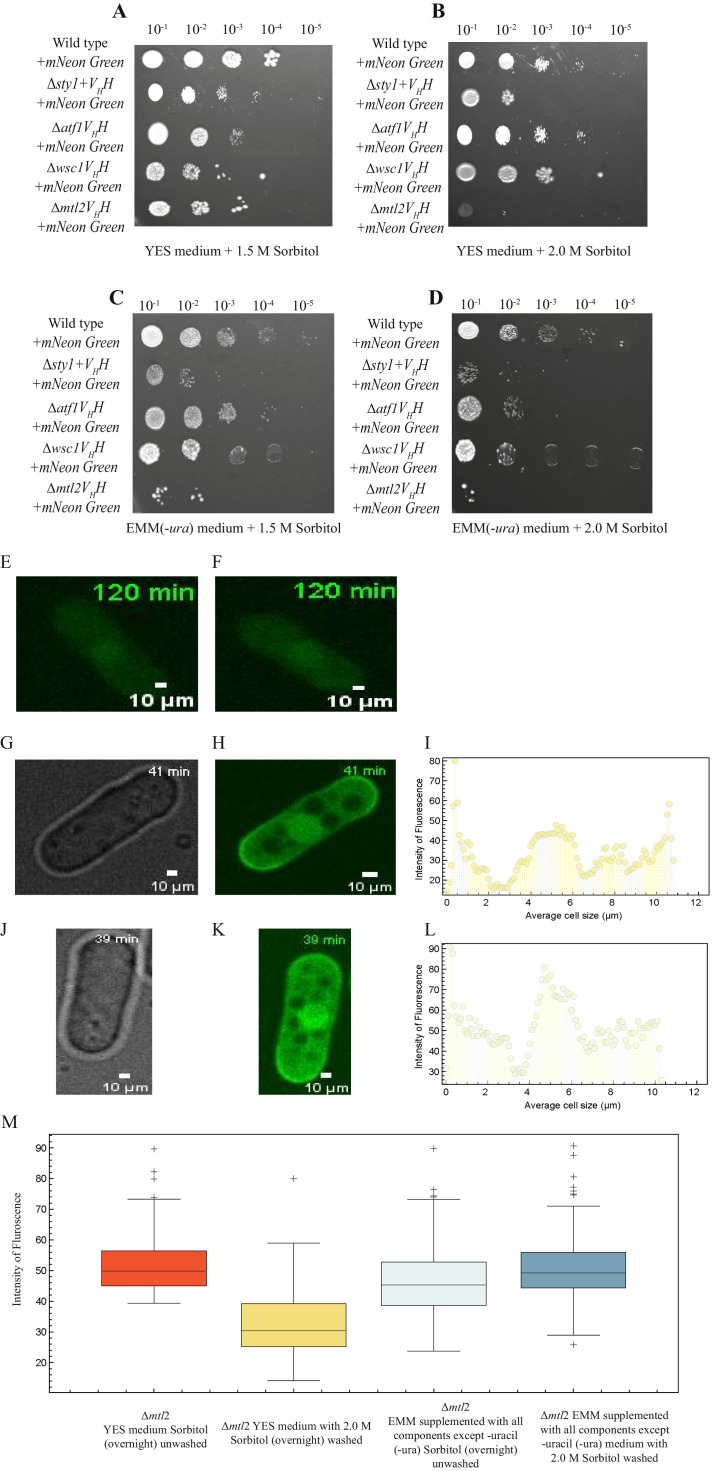
**Spot testing A** and **B** represent YES medium, while **C** and **D**. denote EMM medium of 1.5 M and 2.0 M with sorbitol concentration *S. pombe* utilizing P-36 nanobodies and with deletion mutants of the Δ*sty*1, Δ*atf*1, Δ*wsc*1 and Δ*mtl*2 genes. **E**. YES medium and **F**. EMM supplemented with all components except uracil (-ura) medium subsequent to exposure to 2.0 M sorbitol. G. DIC imaging of Δ*mtl*2 (AL16), **H**. Confocal GFP analysis with P-36 in Δ*mtl*2 (AL16), **I**. Graph depicting fluorescence intensity at the cell terminus following washing with YES medium. **J**. DIC of Δ*mtl*2 (AL16), **K**. Confocal GFP with P-36 in Δ*mtl*2 (AL16), **L**. Graphical representation indicates a considerable increase in fluorescence intensity at the termini of cells in EMM supplemented with all components except uracil (-ura) media. **M. Box plot** illustrating the fluorescence in the presence of a lethal dose of 2.0 M sorbitol in YES medium and EMM supplemented with all components except uracil (-ura) media overnight and compared with the cells which were washed after 30 min with respective fresh media.

The study assessed the sensitivity of various fission yeast mutants, including Δ*sty1*, Δ*atf1*, Δ*wsc1*, and Δ*mtl2*, to different concentrations of sorbitol by spot tests on YES medium. A notable difference was detected in the Δ*mtl2* mutant, which exhibited increased sensitivity to high sorbitol concentrations of 1.5 M (Fig. 3A) and 2.0 M (Fig. 3B Spot Test). Both 1.5 M (Fig. 3C) and 2.0 M (Fig. 3D) concentrations in EMM supplemented with all components except uracil (-ura) medium exhibited fatal sensitivity of Δ*mtl2* at a sorbitol concentration of 2.0 M. Microscopic examination indicated that the yeast exhibited no immediate response to 2.0 M sorbitol (Fig. 3E and F) (Supplementary Videos S9 and S10 at https://doi.org/10.6084/m9.figshare.28756475.v1); however, upon introduction to fresh YES medium, the organism exhibited the formation of actin filaments at the cell termini after approximately 23 min (Fig. 3G, H and I) (Supplementary Video S11 and S12 at https://doi.org/10.6084/m9.figshare.28756475.v1) and with EMM supplemented with all components except uracil (-ura) fluorescence was observed after 39 min (Fig. 3J, K and L) (Supplementary Video S13 and S14 at https://doi.org/10.6084/m9.figshare.28756475.v1) . This indicates that sorbitol exerts an inhibitory effect, which can be alleviated by substituting sorbitol with fresh medium to reestablish a conducive environment. The comparative analysis of fluorescence in Δ*mtl2* under non-stress and stress settings in YES and EMM media supplemented with all components further substantiates the inhibitory action of sorbitol, as indicated by a marked reduction in fluorescence intensity in YES medium under stress conditions (Fig. 3M Box Plot).

#### 3.2.2 Effect of KCl (Potassium chloride)

**Fig. 4:**
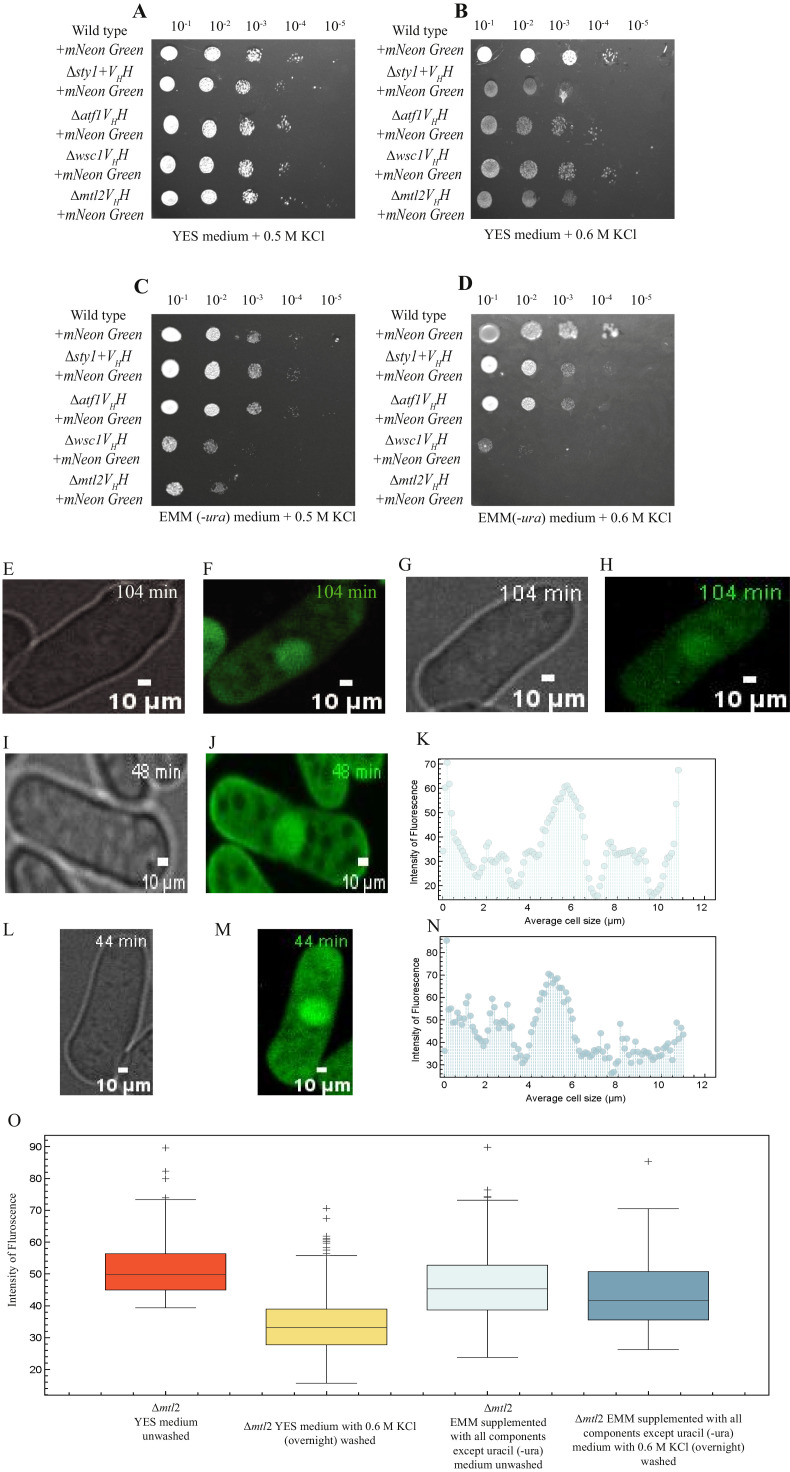
**Spot testing A** and **B**. represent YES medium while **C** and **D**. represent EMM supplemented with all components except uracil (-ura) medium of 0.5 M and 0.6 M with KCl concentration *S. pombe* utilizing P-36 nanobodies and deletion mutants of the Δ*sty*1, Δ*atf*1, Δ*wsc*1 and Δ*mtl*2 genes. **E**. DIC, F. Confocal GFP in YES medium and **G**. DIC, **H**. Confocal GFP in EMM supplemented with all components except uracil medium following exposure to 0.6 M KCl. **I**. DIC of Δ*mtl*2 (AL16), **J**. Confocal GFP with P-36 in Δ*mtl*2 (AL16), **K**. Illustration depicting the fluorescence intensity subsequent to washing with YES medium. **L**. DIC of Δ*mtl*2 (AL16), M. Confocal GFP with P-36 in Δ*mtl*2 (AL16), N. Illustration depicting fluorescence intensity in EMM supplemented with all components except uracil (-ura) media. **O**. Box plot contrasting fluorescence levels with and without a lethal dosage of 0.6 M KCl Sorbitol in YES medium and EMM supplemented with all components except uracil (-ura) media overnight and compared with the cells which were washed after 30 min with respected fresh media.

**Fig. 5:**
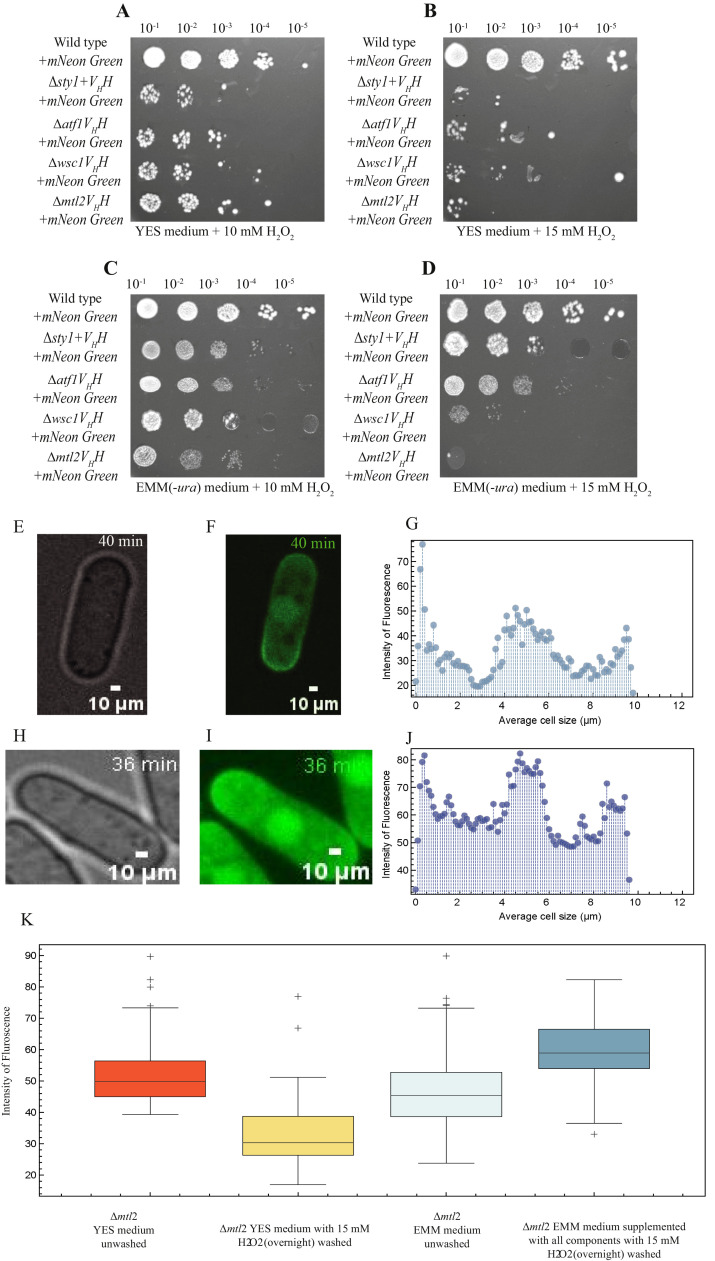
**Spot testing A** and **B.** represent YES media, while **C** and **D**. denote EMM medium supplemented with all components except uracil (-ura) of 10 mM and 15 mM with H_2_O_2_ concentration in *S. pombe* utilizing P-36 nanobodies and with deletion mutants of the Δ*sty*1, Δ*atf*1, Δ*wsc*1 and Δ*mtl*2 genes. **E**. DIC, **F**. Confocal GFP in YES media, **G**. illustration of fluorescence intensity post-washing with YES medium, **H**. DIC, & **I**. Confocal GFP in EMM supplemented with all components except uracil (-ura) media following exposure to 15 mM with H_2_O_2_. **J**. Depiction of the fluorescence intensity subsequent to washing with EMM supplemented with all component’s media. **K**. Box plot illustrating fluorescence level with and without a lethal dose of 15 mM of H₂O₂ in YES medium and EMM supplemented with all components except uracil (-ura) media overnight and compared with the cells which were washed after 30 min with respective fresh media**.**

Potassium chloride (KCl) can be employed in fission yeast (*Schizosaccharomyces pombe*) to investigate osmoregulation, cell cycle progression, or various physiological responses. The deleted mutants were initially evaluated on YES medium by a spot test in the presence of P-36 nanobodies. Δ*sty1,* Δ*atf1,* and Δ*wsc1* exhibited varying sensitivity to P-36 at KCl concentrations of 0.5 M and 0.6 mM. A notable discrepancy was observed in Δ*mlt2* (Fig. 4A and B Spot Test). Comparable outcomes were noted with mutants cultivated on EMM supplemented with all components except uracil (-ura) (Fig. 4C and D Spot Test). The sensitivity indicates that Δ*mlt2* has heightened reactivity to higher concentrations of KCl. The yeast exhibited no reaction when examined microscopically in the presence of 0.6 M KCl (Fig. 4E, F, G, and H). After exposure to YES media, the organism exhibited actin filament synthesis at the cell termini approximately 48 min later (Fig. 5I, J, and K) (Supplementary Videos S15, S16, S17, and S18 at https://doi.org/10.6084/m9.figshare.28756475.v1), while utilizing sorbitol in YES and EMM supplemented with all components except uracil (-ura) medium after around 44 min (Fig. 4L, M, and N) (Supplementary Videos S19, S20, S21, and S22 at https://doi.org/10.6084/m9.figshare.28756475.v1). This indicates that KCl exerts an inhibitory effect, which is alleviated by substituting it with fresh medium, thereby promoting the activation of actin filaments while maintaining the same duration as the microorganism under standard, non-stress conditions. The box plot depicts the comparative fluorescence of Δ*mtl2* in non-stress conditions against Δ*mlt2* in stress conditions inside YES and EMM supplemented with all components except uracil (-ura) medium (Fig. 4O Box Plot). The fluorescence intensity in YES media was markedly reduced; however, in EMM supplemented with all components except uracil (-ura) medium, it was somewhat increased under stress circumstances.

#### 3.2.3 Impact of H_2_O_2_ (Hydrogen peroxide)

The deleted mutants were initially evaluated on YES medium by a spot test in the presence of P-36 nanobodies. Δ*sty*1, Δ*atf*1, and Δ*wsc*1 demonstrated differential sensitivity to 10 mM and 15 mM concentrations of H_2_O_2_ when treated with P-36. A significant mismatch was seen in Δ*mtl*2. At 10 mM, growth was restricted to 10^-4, while at 15 mM, a concentration of 10^-1 proved lethal to the organism. Fig. 5A and B Spot Test. Similar results were observed with mutants grown on EMM supplemented with all components except uracil (-ura) (Fig. 5C and D Spot Test). The sensitivity demonstrates that Δ*mtl*2 has increased reactivity to elevated doses of H_2_O_2_. The yeast displayed no reaction when seen microscopically in the presence of 15 mM H_2_O_2_. Following exposure to YES media, the organism demonstrated actin filament synthesis at the cell termini after approximately 40 min in YES (Fig. 5E, F, and G) and after roughly 36 min in EMM supplemented with all components except uracil (-ura) medium (Fig. 5H, 5I, and 5J) (Supplementary Videos S23, S24, S25, and S26 at https://doi.org/10.6084/m9.figshare.28756475.v1). This suggests that H_2_O_2_ has an inhibitory impact, which is mitigated by replacing H_2_O_2_ with fresh media, thus facilitating the activation of actin filaments while preserving the same timing as the microorganism in standard, non-stress conditions. The box plot depicts the comparative fluorescence of Δ*mlt*2 under non-stress settings (Fig. 5K Box Plot).

#### 3.2.4 Impact of caspofungin (Csp)

**Fig. 6:**
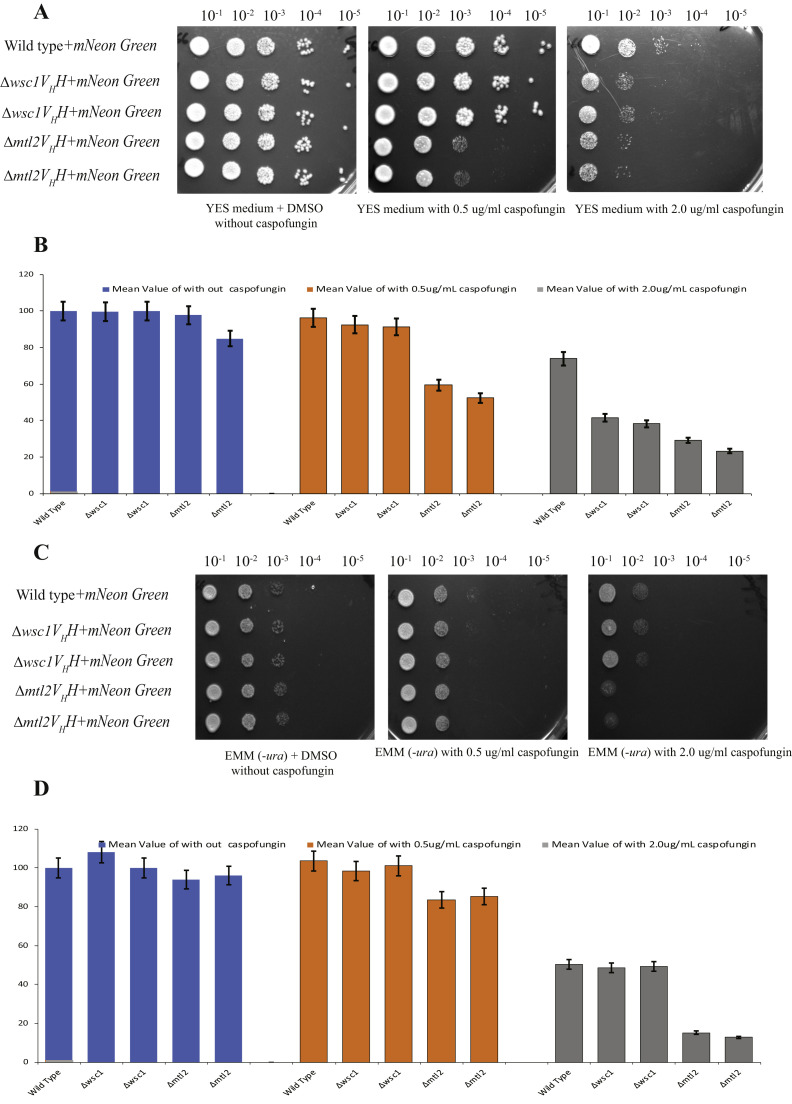
**Spot testing A** & **B** findings demonstrate the sensitivity of Δ*wsc*1 and Δ*mtl*2 at varying concentrations of caspofungin on YES medium. The growth of fission yeast is normal in the presence of DMSO. 0.5 µg/mL of Caspofungin in Δ*wsc*1 and Δ*mtl*2 strains exhibiting diminished growth in Δ*mtl*2. 2.0 µg/mL of Caspofungin Δ*wsc*1 and Δ*mtl*2 strains exhibiting substantial growth reduction for Δ*wsc*1 and Δ*mtl*2. C & D **Spot testing** results findings demonstrating the sensitivity of Δ*wsc*1 and Δ*mtl*2 at varying concentrations of caspofungin on EMM supplemented with all components except uracil (-ura) medium. The growth of fission yeast is normal in the presence of DMSO. 0.5 µg/mL of Caspofungin Δ*wsc*1 and Δ*mtl*2 strains exhibiting diminished growth Δ*mtl*2. 2.0 µg/mL of Caspofungin Δwsc1 and Δ*mtl*2 strains exhibiting a notable reduction in growth for Δ*wsc*1 and Δ*mtl*2. The experiment was performed n = 3 times.

The findings indicate that yeast growth in the presence of Csp (Caspofungin), a lipopeptide antibiotic that inhibits β-(1,3) glucan biosynthesis at concentrations of 0.5 and 2.0 µg/mL, exhibited results akin to the wild type for Δ*wsc*1. In contrast, Δ*mtl*2, tagged with the pDUAL-Pef1a plasmid, demonstrated increased sensitivity relative to the wild type on YES media, as illustrated in (Fig. 6A and 6B Spot test and Graph). We subsequently examined cells harbouring the pDUAL-Pef1a plasmid labelled with the mNeonGreen reporter gene on EMM supplemented with all components except uracil (-ura) at two distinct concentrations of caspofungin. The Δ*mtl*2 strain exhibited reduced sensitivity at 0.5 µg and 2.0 µg/mL of caspofungin in comparison to Δ*wsc*1, as illustrated in (Fig. 6C and D Spot test and Graph). Due to its heightened sensitivity, Δ*mtl*2 was subjected to further investigation for the screening of nanobodies at a sublethal concentration of caspofungin.

### 3.3 Isolation of the plasmid from the yeast organism

**Fig. 7:**
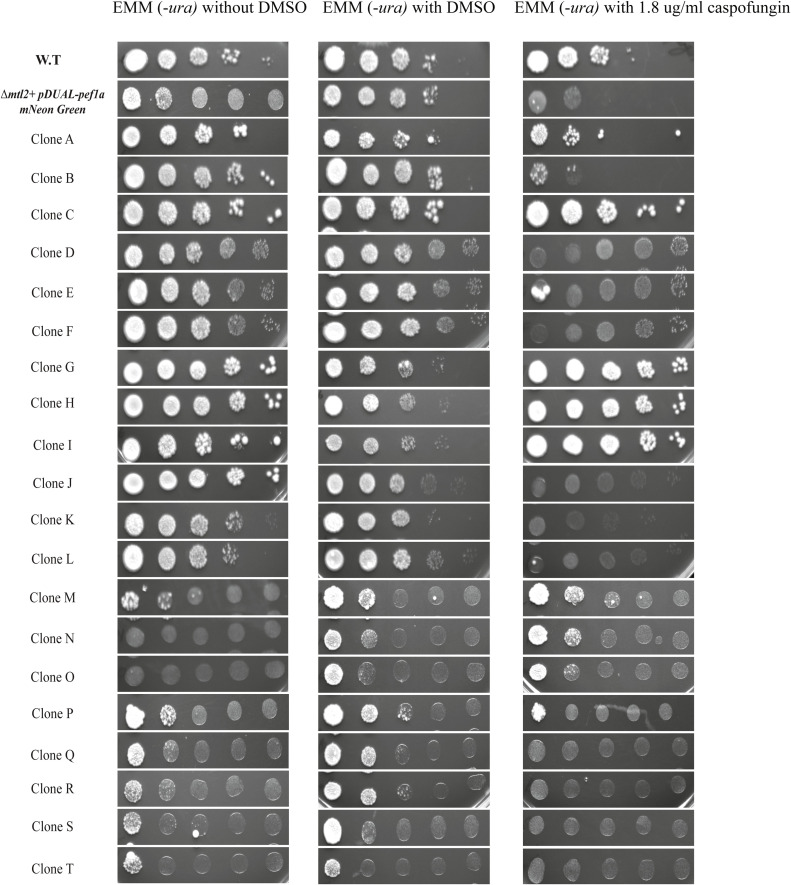
**Spot testing** for all isolated clones following the transformation of V_H_H nanobodies into Δ*mtl*2 recipient cells. Subsequently, plasmid extraction was performed using a genomic extraction kit, and the purified plasmid was introduced into the Δ*mtl*2 strain recipient cells.

Each clone was separately grown in EMM supplemented with all components except uracil (-ura) broth supplemented with hygromycin overnight. The yeast genome was isolated using a genome isolation kit and subsequently amplified using PCR with forward and reverse primers of the pDUAL-Pef1a plasmid preceding mNeonGreen, as illustrated in (Fig. 7 Spot Test). The 369 bp band size was partitioned and allocated into two segments on the gel. One sample was ligated into the *Bam*HI and *Hind*III-digested pDUAL-Pef1a plasmid and subsequently transformed into Δ*mlt*2 cell, while the second sample was dispatched for nucleotide sequencing. Twenty distinct isolates featuring randomized CDR regions were obtained. They were organized in alphabetical order based on their isolation.

### 3.4 Imaging and fluorescent microscopy

**Fig. 8:**
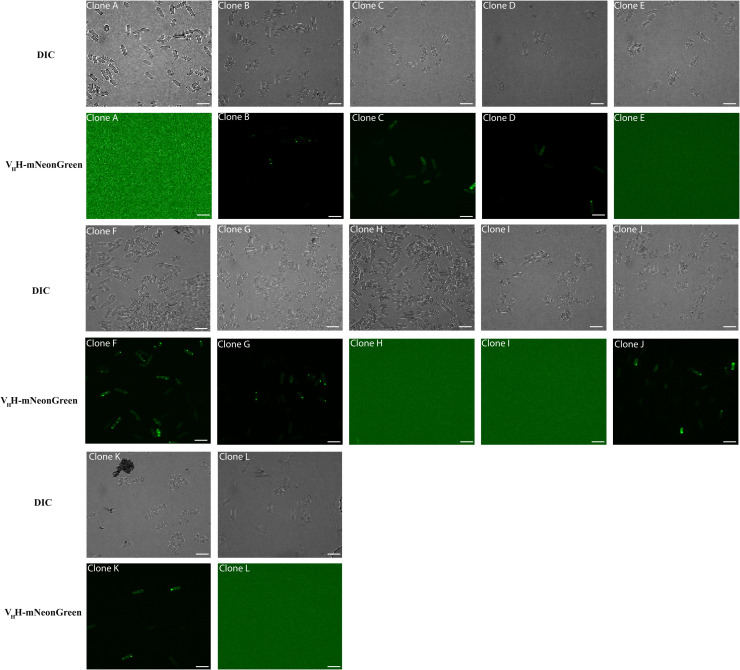
The clones isolated following the transformation of the V_H_H library on caspofungin EMM supplemented with all components except uracil (-ura) plates at a concentration of 1.8 µg/mL are depicted. Clones B, C, D, F, G, J & K exhibited fluorescence localization and facilitated cell recovery under stress conditions, whereas clones A, E, H, I & L displayed no fluorescence. The specific CDR domain on V_H_H responsible for antigen binding which conferred resistance to the organism against caspofungin would be identified. The fluorescence was observed in the cytoplasm and was more consistent in Clone-C.

**Fig. 9:**
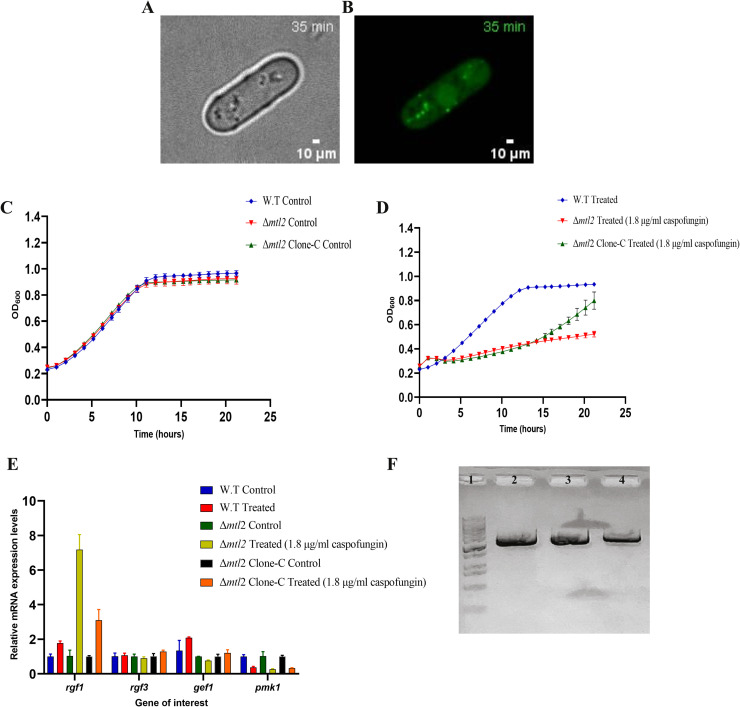
**A**. Differential Interference Contrast (DIC) & **B**. Confocal Green Fluorescent Protein (GFP) in EMM supplemented with all components except uracil (-ura) media containing 1.8 µg/mL Caspofungin. **C**. Analysis of Growth Curve: EMM (supplemented with all component’s except uracil (-ura) Normal exhibiting wild type (AL06) and Δ*mtl*2 (AL15) and Δ*mtl*2 with VHH (Clone-C) **D**. EMM supplemented with all components except uracil (-ura) 1.8 μg/ml Caspofungin wild type (AL06) exhibits growth comparable to that without caspofungin whereas Δ*mtl*2 (AL15) attains on OD_600_ 0.5 at a significantly slower rate compared to Δ*mtl*2 with V_H_H (Clone-C) (ANOVA, p = 0.09814) **E**. qPCR expression study indicates that the presence of nanobody *rgf*1 is diminishes cell survival, whereas in the absence of the nanobody in Δ*mtl*2, its expression is dramatically elevated with n = 3 samples repeated 3 times. **F. Western blot analysis**: 1. Western blot analysis: **1. Leader 2**. Δ*mtl*2 in the presence of V_H_H. The expression of Rgf1 was significantly increased. **3**. Wild type treated with caspofungin. The standard bandwidth expression of RGF1.**4**. Δ*mtl*2 was subjected to caspofungin treatment. The expression of Rgf1 is drastically reduced.

We transformed and isolated of the pDUAL-Pef1a plasmid in Δ*mtl*2 recipient cells. The pDUAL-Pef1a plasmid, containing nanobodies, was conjugated with mNeonGreen fluorescence. Each clone was examined using a fluorescence microscope. Seven fluorescent clones were identified: Clone B, C, D, F, G, J, and K (Table 4) (Fig. 8 Selected clones) Clone A, H, I, Q and R did not show any fluorescence. The sequence of Clone-G was similar to Clone-C. The underwent spot testing following the rebuilding of the new pDUAL-Pef1a plasmid in fission yeast cells. The Clone-C of Δ*mtl*2 was utilized for fluorescent confocal microscopy to investigate plasmid localization, which was found to be in the cytoplasmic region of the cell. (Fig. 9A and B Confocal GFP microscopy) (Supplementary Videos-S27 and S28 at https://doi.org/10.6084/m9.figshare.28756475.v1).

**Table 4:** CDR Sequence of the isolated clones with pDUAL-Pef1a plasmid in Δmtl2.

Clone Identification	CDR DNA sequences in pDUAL-Pef1a plasmid along with the possible amino acid sequence	Presence of Fluorescence
**Clone-B**	CDR1 AATGTTCTTACGAAGATTGAG (MFLRRL)(Met, Phe,Leu,Arg,Arg,Leu) CDR2 GAGAGTGCTTCTATTCCGGAG (ESASIPE) (Glu, Ser, Ala, Ser, Ile, Pro, Glu) CDR3 GACTGAGTCGCAGAATTATAAGCGTTAT (D-VAEL-AL) (Asp, stop, Val, Ala, Glu, Leu, Stop, Ala, Leu)	Green Fluorescent
**Clone-C**	CDR1 CATGCTGATCAGATGCCTACT (HADQMPT) (His, Ala, Asp, Gln, Met, Pro, Thr) CDR2 GCTAGGAGGATTTTGTTTTCT (ARRILFS) (Ala, Arg, Arg, Ile, Leu, Phe, Ser) CDR3 AATCATCATGTGTTTCTTGTGAAGACT (NHHVFLVKT) (Asn, His, His, Val, Phe, Leu, Val, Lys, Thr)	Green Fluorescent
**Clone-D**	CDR1 GATCAGCCTCGTTCTCCGATT (DQPRSPI) (Asp, Gln, Pro, Arg, Ser, Pro, Ile) CDR2 TGTACGCATCCTTTTTTGGC (CTHPFL) (Cys, Thr, His, Pro, Phe, Leu) CDR3 TCTACGAGTGTGTCTCCTAATCATTGT (STSVSPNHC) (Ser, Thr, Ser, Val, Ser, Pro, Asn, His, Cys)	Green Fluorescent
**Clone-E**	CDR1 TTTTTTTCTGTTGTTTTTACT (FFSVVFT) (Phe, Phe, Ser, Val, val, Phe, Thr) CDR2 TCTGATAATGTGATGGATACG (SDNVMDT) (Ser, Asp, Asn, Val, Met, Asp, Thr) CDR3 ACTAATACTAGGCAGGAGCCGTATCTG (TNTRQEPYL) (Thr, Asn, Thr, Arg, Gln, Gln, Pro, Tyr, Leu)	No Fluorescent
**Clone-F**	CDR1 AATTGGCATGGGGTGCTGGGT (NWHGVLG) (Asn, Trp, His, Gly, Val, leu, Gly) CDR2 CTTTGTATGAATCATCTGCTT (LCMNHLL) (Leu, Cys, Met, Asn, His, Leu, Leu) CDR3 TTGTTGAATGGGGGTAGGTCTACTAGG (LLNGGRSTR) (Leu, leu, Asn, Gly, Gly, Arg, Ser, Thr, Arg)	Green Fluorescent
**Clone-J**	CDR1 GGTGAGTGGAAGGCGCTTCAG (GEWKALQ) (Gly, Glu, Trp, Lys, Ala, Leu, Gln) CDR2 GGTAGTAATGCTCGGGCTGAT (GSNARAD) (Gly, Ser, Asn, Ala, Arg, Ala, Asp) CDR3 CCGCCTTGGCATTGGGCTTTTCGGATG (PPWHWAFRM) (Pro, Pro, Trp, His, Trp, Ala, Phe, Arg, Met)	Green Fluorescent
Clone-K	CDR1 GATTGGCGTGATCTTTATTAG (DWRDLY-) (Asp, Trp, Arg, Asp, Leu, Tyr, stop) CDR2 AGTTTTCAGTCTGGGAGGTAT (SFQSGRY) (Ser, Phe, Gln, Ser, Gly, Arg, Tyr) CDR3 CTGTAGAATTCTCTTTTTGATTTTCCG (L-NSLFDFP) (Leu, Stop, Asn, Ser, leu, Phe, Asp, Phe, Pro)	Green Fluorescent
Clone-L	CDR1 TGTAGTTCTAAGACGTATATG (CSSKTYM) (Cys, Ser, Ser, Lys, Thy, Tyr, Met) CDR2 GAGCTTGGTCCGGATGCTTT (ELGPDA) (Glu, Leu, Gly, Pro, Asp, Ala) CDR3 TTGTAGAATGTTGCGTATCTGAAGAAG (L-NVAYLKK) (Leu, Asn, Val, Ala, tyr, Leu, Lys, Lys)	No Fluorescent
Clone-N	CDR1 GGAACGGGTGGTCGTCTTTGG (L-NVAYLKK) (Leu, Asn, Val, Ala, Tyr, Leu, Lys, Lys) CDR2 CGGGCTCGTCCGGCTGTGGTGG (RARPAVV) (Arg, Ala, Arg, Pro, Ala, Val, Val) CDR3	No Fluorescent
Clone-O	CDR1 GTGCATCTTGCTATTCCTCAT (VHLAIPH) (Val, His, Leu, Ala, Ile, Pro, His) CDR2 GCGAAGGGGTGGTGTGTGCTT (AKGWCVL) (Ala, Lys, Gly, Trp, Cys, Val, Leu) CDR3 ATGATTCATTTGGGGAATGTTCTTTGG (MIHLGNVLW) (Met, Ile, His, Leu, Gly, Asn, Val, Leu, Trp)	No Fluorescent
Clone-P	CDR1 GCTCCTAGTATTGATAATCCG (APSIDNP) (Ala, Pro, Ser, Ile, Asp, Asn, Pro) CDR2 AAGAAGCATAAGATGGGTGTG (KKHKMGV) (Lys, Lys, His, Lys, Met, Gly, Val) CDR3 TGGGATAATGCTGTGATTCAGGGGACG (WDNAVIQGT) (Trp, Asp, Asn, Ala, Val, Ile, Gln, Gly, Thr)	No Fluorescent
Clone-S	CDR1 TTGGATTCTTCTTCTCATATT (LDSSSHI)(Leu, Asp, Ser, Ser, Ser, His, Ile) CDR2 GGGCGGTTGTGGGGCGGTTG (GRLWGG) (Gly, Arg, Leu, Trp, Gly, Gly) CDR3 GTGGTGTTTGGGGGTGAGGCTTCGTTT (VVFGGEASF) (Val, Val, Phe, Gly, Gly, Glu, Ala, Ser, Phe)	No Fluorescent
Clone-T	CDR1 CCTCCTTCGCATCTGATTACT (PPSHLIT) (Pro, Pro, Ser, His, Leu, Ile, Thr) CDR2 ACTCAGCCTCTGAAGAAGAAT (TQPLKKN) (Thr, Gln, Pro, Leu, Lys, Lys, Asn) CDR3 CTTGATGAGCGGAATCATTAGTAGGTG (LDERNH–V) (Leu, Asp, Glu, Arg, Asn, His, Stop, stop, Val)	No Fluorescent

### 3.5 Analysis of growth curves

The growth assessment of Clone-C (which includes nanobodies and mNeonGreen) indicates diminished growth compared to the empty pDUAL-Pef1a plasmid (lacking nanobodies). The results show that the nanobody sequence facilitates the growth yeast cells despite exposure to a lethal dose of caspofungin at 1.8 µg/mL (Fig. 9C and D Growth Curve). Our aim is to identify genes that exhibit alteration in expression at the molecular level.

### 3.6 Quantitative polymerase chain reaction (qPCR) analysis

The data indicate that *rgf*1 expression was markedly elevated in organisms possessing the pDUAL-Pef1a plasmid devoid of the nanobody sequence, whereas in the strain containing the pDUAL-Pef1a plasmid with nanobodies, *rgf*1 levels were considerably diminished. Rgf1 demonstrates a specific association with the absence of Mtl2, consequently augmenting the microorganism's resilience in the presence of caspofungin. Conversely, *rgf*1 expression diminished in Δ*mtl*2 cells with nanobody-expressing plasmids, although levels were still elevated compared to WT (Fig. 9E qPCR expression). The data indicate that without Mtl2, nanobodies may influence Rgf1 activity, thus improving cell survival during caspofungin-induced stress.

### 3.7 Western blotting

A Western blot was conducted on total extracts to assess total RgF1 levels (whole cell extracts). The units denote the variation in Rgf1 expression, corroborating the qPCR findings. The wild type, lacking the Δ*mtl*2, exhibited normal expression levels, whereas the Δ*mtl*2 showed a substantial reduction in Rgf1 expression. In clone-C cells containing pDUAL+V_H_H+linker+mNeonGreen, the expression level was markedly elevated in the presence of caspofungin. It suggests that V_H_H influences Rgf1 to facilitate cellular survival Fig. 9F.

### 3.8 Dynamics and structural analysis of the rgf1 binding domain

Anticipating protein-protein interactions is crucial for understanding cellular function and organisation. The mechanistic analysis of interactions frequently requires comprehensive chemical data, usually obtained using X-ray crystallography or nuclear magnetic resonance (NMR). Nevertheless, numerous biologically relevant interactions happen in temporary groups, making it harder to figure out their structures through experiments, as shown by *rgf*1 and V_H_H (Clone-C) from *S.pombe*. The computational docking technique facilitates the recognition of complicated structures that are not readily understandable. These predictions allow us to determine the probability of contact between two molecules. This experiment highlights the amino acids from the CDR1, CDR2, and CDR3 regions that directly engage with the Rgf1 protein molecule. The Rgf1 protein model was acquired from the AlphaFold website (sequence code = AF-Q9Y7U6-F1). After assessing the V_H_H (Clone-C) 3D model in comparison to Rho1, we acquired 24 predictions from the HADDOCK website for the root mean square deviation (RMSD), from which we selected the model exhibiting the lowest energy (0.9 ± 0.8). (**Supplementary Figure S6)** was chosen for further observation and analysis. The CDRs form significant binding contacts with the Rgf1 binding domain. There exist seven interaction sites in all.

The bond distances between the CDRs and Rgf1 (see **Supplementary Table S4**) show that the amino acid Glutamine 104, interacts the most with Rfg1, connecting with Rho1 at Histidine 106 (2.775 Å), Serine 109 (2.718 Å), and Leucine 105 (2.858 Å). Notably, none of the binding domains of the CDRs engaged with the active protein sites, including Rho1 G15 (Glycine), Q64 (Glutamine), D121 (Aspartic acid), T20 (Threonine), and C199 (Cystine). Rgf1 is crucial for the localisation of actin patches at their apices, thereby enhancing cell polarity, which subsequently aids in the formation of cell morphology and septation post-elongation.

## 4 Discussion

Nanobody-Mediated Regulation of the Mtl2–Rgf1–Rho1GTPase Pathway: In-Depth Examination

This study associates stress-sensitive phenotypes of *mtl*2-deficient fission yeast with an in vivo nanobody screening, identifying Rgf1 as a vital component affected by a particular nanobody to improve survival during lethal caspofungin exposure. The results integrate phenotypic stress testing, nanobody selection, expression analysis, and structural modelling into a unified regulatory framework [7,37]. The capacity to rapidly and reversibly eliminate target proteins, including concurrent combinatorial depletion, underscores their versatility in examining complex biochemical processes inside this model organism [38]. Moreover, exploring inducible nanobody expression systems would provide temporal control of protein disruption, offering an advanced approach to investigate dynamic biological processes and mitigate potential adaptive responses [39,40]. The continuous progress of nanobody technology in fission yeast will facilitate the comprehensive examination of low-abundance proteins within their native cellular environment, a significant challenge for traditional methods [41]. Differential stress sensitivity and the crucial role of *mtl*2: Under osmotic (sorbitol, KCl) and oxidative (H₂O₂) stressors, Δ*mtl*2 is consistently the most sensitive mutant, demonstrating "fatal sensitivity" at high sorbitol concentrations and marked inhibition by KCl and H₂O₂, with no immediate actin-based response until the stress is mitigated and the organism is transferred to fresh medium. A similar delayed recovery is noted after exposure to KCl and H₂O₂, where actin reappears at the tips immediately upon replacement with a non-stress solution, with time equivalent to that of unstressed cells. These findings highlight Mtl2 as an essential cell wall sensor required for the swift Rho1-dependent reestablishment of polarity after diverse stresses. In the presence of caspofungin, Δ*mtl*2 demonstrates heightened sensitivity compared to the wild type on YES at concentrations of 0.5–2.0 µg/mL, suggesting impaired cell-wall integrity, while the composition of the medium and plasmid selection affect the relative sensitivity in comparison to Δ*wsc*1 on EMM enriched with all components except -uracil. The integration of spot tests and live imaging supports a model in which Mtl2 is crucial for the initiation and sustenance of cell-wall stress responses, exceeding Wsc1 under the conditions studied. The administration of antifungal agents induces damage, necessitating the repair and regeneration of the cell wall via biosynthesis. Recent studies have focused on the structure and function of the yeast cell wall [42,43].

In Vivo Screening and Identification of Protective Nanobody Variants: The transformation of a synthetic V_H_H library on Δ*mtl*2 yielded a significant quantity of transformants, indicating effective library coverage and appropriateness for nanobody screening in *S. pombe*. Selection at a lethal dosage of caspofungin (1.8 µg/mL) produced clones that survived and expressed mNeonGreen-tagged nanobodies; seven displayed significant fluorescence (B, C, D, F, G, J, and K), with Clone-C showing robust, homogeneous cytoplasmic localisation.

Growth curves demonstrate that Δ*mtl*2 with Clone C nanobody shows markedly improved growth relative to Δ*mtl*2 with an empty vector at 1.8 µg/mL caspofungin, approaching wild-type growth, but there is a slight reduction in basal growth due to nanobody expression. Thus, the selection of nanobodies under stress effectively identifies functional binders that alter signalling pathways to improve survival.

RGF1 as a Primary Effector of Nanobody-Mediated Restoration The qPCR analysis reveals that *rgf*1 is significantly overexpressed in Δ*mtl*2 with the empty pDUAL-Pef1a under caspofungin treatment; however, Δ*mtl*2 with nanobody-expressing plasmids exhibits a marked decrease in *rgf*1 transcripts, although levels remain elevated relative to the wild type. This suggests that the depletion of Mtl2 triggers maladaptive upregulation of *rgf*1, which is partially corrected by V_H_H expression. The monovalent nature of nanobodies allows for the addition of fluorescent tags without compromising their binding efficiency, a significant advantage over scFvs, where tagging often affects affinity [44]. Western blotting provides additional evidence at the protein level: total Rgf1 is "substantially" or "drastically" reduced in Δ*mtl*2, while Δ*mtl*2 Clone-C cells demonstrate significantly elevated Rgf1 expression in the presence of caspofungin, consistent with the stabilisation or enhanced production of Rgf1 resulting from V_H_H binding. The reinstatement of Rgf1, recognised for its function in actin patch localisation, polarity, and septation, likely explains the improved formation and reemergence of tip-localised actin under stress. Docking analysis improves the mechanistic model. HADDOCK delineates seven contact sites between Clone-C CDRs and Rgf1, with Glutamine 104 forming many close associations; however, none of the CDR interactions involve the recognised active residues of Rho1 (G15, Q64, D121, T20, and C199). The data suggest a modulatory interaction that stabilises or optimises Rgf1 conformation rather than hindering its catalytic interface, aligning with the observed gain-of-function rescue.

The molecular data confirm a Mtl2–Rgf1–Rho1GTPase axis: the lack of Mtl2 disturbs Rgf1 expression and levels, hindering Rho1 activation and cell wall repair; a nanobody directed at Rgf1 restores Rgf1 levels and promotes recovery. Rho1-facilitated survival under echinocandin-induced stress.

The inherent stability and versatility of nanobodies make them especially appropriate for many fluorescence-based applications, including high-resolution imaging and real-time cellular monitoring [45]. Furthermore, the small size of nanobodies facilitates rapid tissue penetration and clearance, which is particularly beneficial for in vivo imaging applications that need minimal background signal and swift target identification [44,46]. The accelerated pharmacokinetics, coupled with their elevated specificity, provide enhanced signal-to-background ratios in diagnostic imaging, hence augmenting the identification of disease-associated antigens [44,47]. RhoGTPase-specific nanobodies demonstrate exact contact durations and can aid in defining the stages of the cell cycle.

*mtl*2 is crucial for preserving the integrity of the cell wall [2,48]. Mtl2 is a constituent of the Rho1/Pck1 signalling pathway, as corroborated by other sources (Supplementary Figure S6). The cells exhibit a morphology akin to that of Rho1 and PcK1 [48,49]. Moreover, Δ*mtl*2 cells exhibit heightened sensitivity to cell wall antifungal agents, a hypersensitivity that is mitigated by the moderate overexpression of *rho*1 [50,51].

**Fig. 10:**
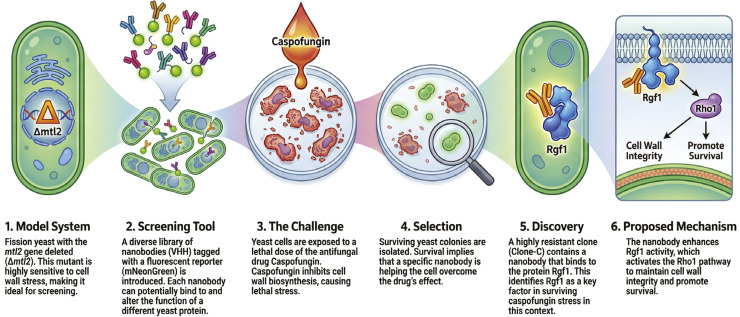
An illustration showing the model and significance of our research in a stepwise manner, explaining how nanobodies can be a useful tool in identifying important functions in the cell.

Methodological and Therapeutic Implications: The study methodically validates in vivo nanobody-library screening in *S. pombe* through pDUAL-Pef1a, chromosomal integration, and fluorescent tagging. This approach bypasses animal vaccination, uses synthetic CDR-diversified V_H_H libraries, and directly correlates survival under particular stress conditions with the selection of functional nanobodies. (Fig. 10) The genetically encoded and fluorescently tagged nanobodies provide straightforward localisation and structural modelling.

The identification of an Rgf1-modulating nanobody that enhances survival during caspofungin therapy highlights Rgf1 and its regulatory counterparts as potential antifungal targets. The authors suggest that the platform can be augmented to incorporate ADC (antibody-drug conjugate) screening, employing nanobodies as targeting agents for critical stress-response components. Subsequent investigations should include other stressors and signalling mutations, directly measure Rho1GTPase activity, and examine the temporal dynamics of nanobody–Rgf1–Rho1 GTPase interactions. Nevertheless, the current findings provide solid evidence for nanobody-mediated gene identification in fungal stress biology.

## Ethics approval

The authors possess authorisation to utilise all photos in the article and have accurately thanked all sources while acknowledging all contributors. This paper has not been published or submitted for publication to any other journal in any format or language. There was no human or animal involved in this study.

## Funding

The author(s) did not receive any financial assistance for the research, authoring, or publishing of this work.

## CRediT authorship contribution statement

**Noureen Rasheed:** Data curation. **XiZe Ali:** Data curation. **Tariq Ali:** Software. **Faiz Muhammad:** Formal analysis. **Muhammad Ali Rasheed:** Writing – review & editing, Writing – original draft, Supervision, Conceptualization.

## Declaration of competing interest

The authors declare that they have no known competing financial interests or personal relationships that could have appeared to influence the work reported in this paper.

## Data Availability

The data that support the findings of this study are openly available in figshare at https://doi.org/10.6084/m9.figshare.28756475.v1. Nanobody-based gene identification of the stress response in fission yeast RhoGTPase, with a focus on the *mtl*2 gene.

## Appendix Supplementary materials

Image, application 1
