# Supplementary material for: Nanobody-based analysis of RhoGTPase stress response in Schizosaccharomyces pombe: Role of mtl2
Source: Biotechnol Rep (Amst). 2026 May 26;51:e00963. doi: 10.1016/j.btre.2026.e00963 (PMC13254718; doi:10.1016/j.btre.2026.e00963)
Supplement: Supplementary file 1 [file mmc1.pdf]

Nanobody-based analysis of RhoGTPase stress response in *Schizosaccharomyces pombe*: Role of *mtl2*

Noureen Rasheed[1,3,4], XiZe Ali[2,3,4], Tariq Ali[3], Faiz Muhammad[4], Muhammad Ali Rasheed[2*]

[1]Department of Biochemistry, University of Karachi, Karachi, Pakistan,

noureenrasheed24@gmail.com. https://orcid.org/0009-0000-8527-7057

[2] Department of Microbiology, University of Karachi, Karachi, Pakistan,

XizeAli032022@gmail.com, https://orcid.org/0009-0008-3644-6123

[3]Department of Pharmaceutics, Faculty of Pharmaceutical Sciences, DOW University of Health Sciences, 74200 Karachi, Pakistan tariq.ali@duhs.edu.pk, https://orcid.org/0000-0002-6310-6983.

[4]Department of Microbiology, Faculty of Life Sciences and Informatics, Balochistan University of Information Technology, Engineering and Management Sciences BUITEMS, 87300, Quetta, Balochistan, Pakistan, faiz.muhammad2@buitms.edu.pk , https://orcid.org/0000-0002-0657-9004.

[*]**Corresponding Author:**

Muhammad Ali Rasheed

Department of Microbiology, University of Karachi, Karachi, Pakistan,

malirasheed2004@gmail.com, ORCID ID: 0009-0003-3231-3714

Supplementary materials

Supplementary Figure

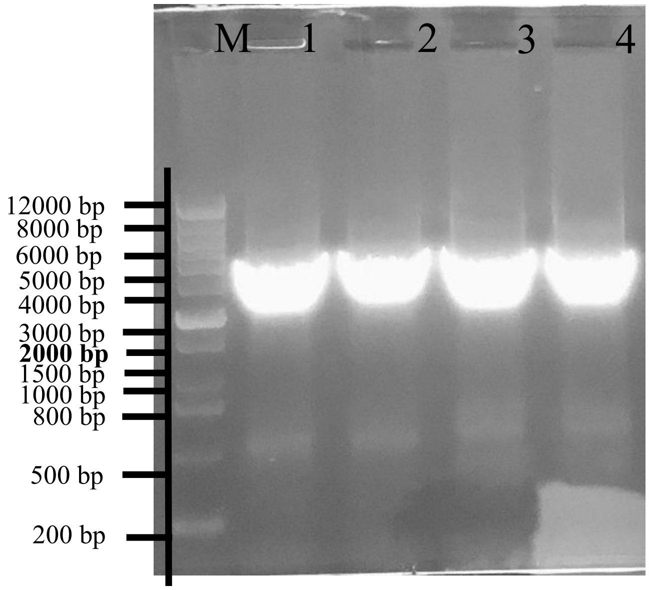

Supplementary Figure S1 For the construction of Δ*sty*11-2 - Amplified *sty1* (5'UTR+hygromycin+3'UTR) in BlueScript SK II for Δ*atf* 3-4 - Amplified atf1 (5'UTR+hygromycin+3'UTR) in BlueScript

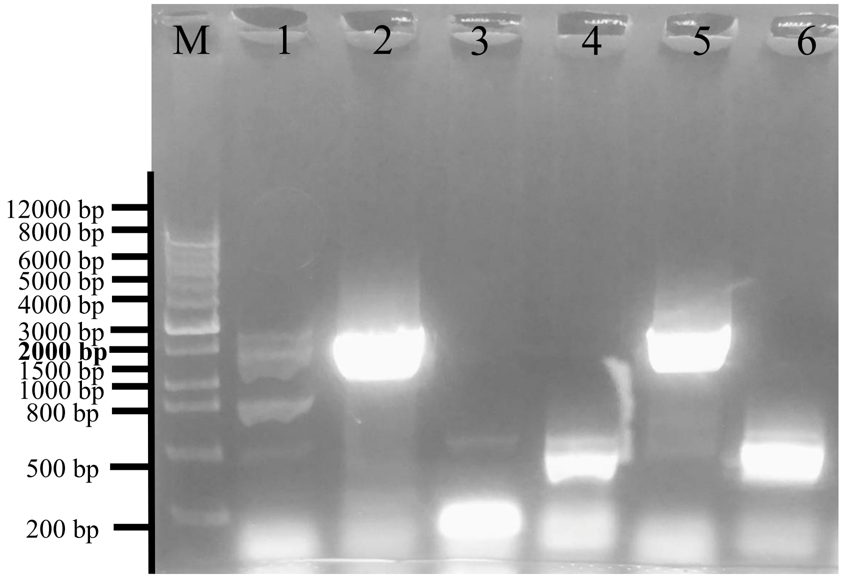

Supplementary Figure S2 1-Amplified using up stream gene of *sty1* Forward Primer and hygromycin gene Reverse Primer in Wild type fission Yeast to confirm the presence of *sty* gene.2- Amplified using Forward Primer up stream gene of *sty1* and Reverse Primer of hygromycin in Δ*sty*1 genome confirming the absence of *sty1* gene.4-Amplified using up stream gene of *atf1* Forward Primer and hygromycin gene Reverse Primer in Wild type fission Yeast to confirm the presence of *atf1* gene.5- Amplified using Forward Primer up stream gene of *atf1* and Reverse Primer of hygromycin in Δ*atf1* genome confirming the absence of *atf1*gene.

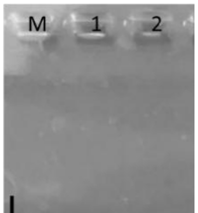

Supplementary Figure S3 1 and 2**-** Amplified using upstream Forward Primer for *mtl*2 and Reverse Primer of hygromycin after isolating genome from Δ*mtl*2. The size of the band was 925 bases confirming the absence of *mtl*2 gene in the genome of fission yeast cell.

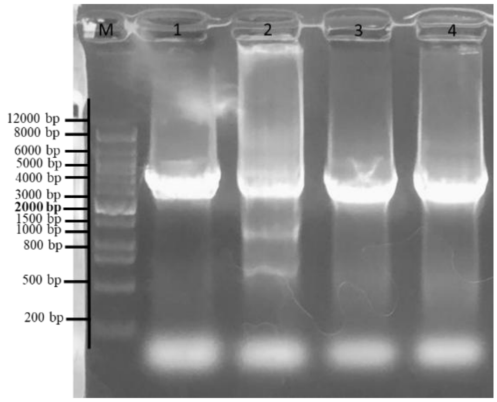

Supplementary Figure S4*:* 1 to 4 **-** Amplified using upstream Forward Primer and hygromycin Reverse Primer after isolating genome from Δ*wsc*1. The size of the band should be 1499 base pair while the current band size was near 2500 bands.

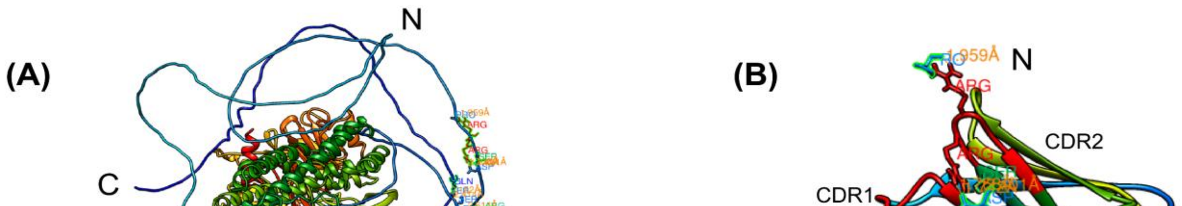

Supplementary Figure S5*:* Protein-Protein interaction between Rgf1 GTPase and V_HH using Chimera, and using website (*https://wenmr.science.uu.nl/haddock2.4/*). A protein-protein docking tool, web site predicted 24 models out of which model 5.1 had the lowest kinetic energy calculated was 0.9 +/- 0.8

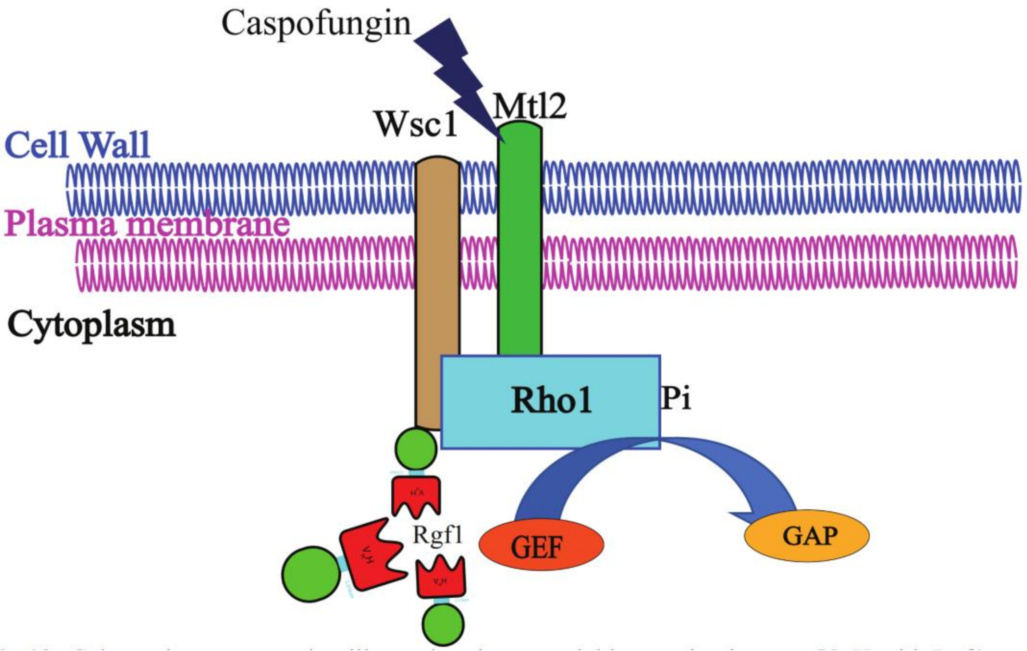

Supplementary Figure S6: Schematic representation illustrating the potential interaction betweenV_HH with Rgf1, resulting in enhanced expression that promotes the survival of yeast cells under elevated concentrations of caspofungin.

Supplementary Tables

Reagents and Resources

Supplementary Table S1

Description	Source	Identifier
Yeast extract	(Sangon Biotech)	CAS #8013-01-2
Glucose	SCR	Cat # 10010518
L-Leucine	Macklin	Lot # C10109207
L-Histidine	Macklin	Lot # C10111685
L-lysin HCl	Macklin	Lot # C10091382
Adenine	Macklin	Lot # C10129555
Uracil	Macklin	Lot # C10136649
Polyethyleneglycol (PEG4000)	Solarbio	CAT # P8240
Lithium acetate dihydrate	Sigma-Aldrich	Lot # SLCC6207
10X TE Buffer (pH7.5)	Shanghai maokang Biotechnology Co. ltd	CAT# MS3541
Sorbitol	Macklin	Lot#C10650838
Yeast peptone Dextrose Agar	Solarbio	Cat#LA0220
5'admentyl-IAA	TCI	CAS 2244426-40-0
Glutamate	Sangon Biotec shanghai	CAS 142-47-2
Seamless Cloning Kit	Bevotime	CAT#D7010M
Hydrogen Peroxide	Macklin	H792077
Potassium Chloride	Macklin	CAT# 104938
Hygromycin B	Sigma-Aldrich	Cat# SBR00039
Ampicillin	Sigma-Aldrich	Cat# A5354
HEPES buffer	GIBCO	Cat# 15630080
Trimethylamine	Sigma-Aldrich	Cat# 471283
b-Mercaptoethanol	Sigma-Aldrich	Cat# 444203
DTT	Thermo Scientific	Cat# R0861
RNAlater solution	Invitrogen	Cat# AM7020

Kits

Description	Source	Identifier
Plasmid isolation kit	TIANprep Mini Plasmid Kit	Cat#DP103-03
Yeast Genome extraction kit	TIANprep Yeast DNA Kit	Cat#DP307-02

FastPure Cell/Tissue Total RNA Isolation Kit V2	Vazyme	
Anti Rabit *rgf*1 antibodies	Received from Dr. Faiz as gift	

Software and Algorithms

Description	Source	Identifier
GraphPad Prism	https://www.graphpad.com/scientific-software/prism/	Version 9.0.0
R-Studio	https://www.rstudio.com/	2021.09.0
Adobe Illustrator	https://www.adobe.com/cn/products/illustrator.	Version 2020 24.0.1
Adobe Photoshop	https://www.adobe.com/cn/products/photoshop/	Version 21.0.2
ImageJ	https://imagej.en.softonic.com/download	ImageJ 1.53c
Snapgene	https://Snapgene.com	Version 4.2.4
MEGA-7	https://www.megasoftware.net/	Version 7
Chimera	https://www.cgl.ucsf.edu/chimera/	Alpha version 1.14
Cluspro	https://cluspro.org/login.php	Kozakov et al., 2017
Ligplot	https://www.ebi.ac.uk/thornton-srv/software/LIGPLOT/	Version 1.4.5
Alphafold	https://alphafold.ebi.ac.uk/	Jumper et al., 2021
Sppider	http://sppider.cchmc.org/	Porollo et al., 2012
Expasy	https://web.expasy.org/translate/	Schwede et al., 2003

List of Oligonucleotides used

Supplementary Table S2

Description	Sequence (5’-3’)
Forward primer of pDUAL-pef1a confirmation	caaacaaaattcgttcgctgatagg
Reverse primer of pDUAL-pef1a confirmation	tacctgagaaagcaacctgacctac
Forward Primer for V_HH in pDual-Pef1a	ctgtttcatcgaactcacgctagcATGGAAGTCCAGCTGCAGGCGTCCG
Reverse Primer for mNeonGreen in pDual-Pef1a	atttagaagtggcgcgccggatccTTActtgtacagctcgtccatgccc
Reverse Primer for in pDUAL-V_HH+linker+mNeon plasmid having linker in the middle vector used pDUAL-pef1a	caccatCGCTCCTGCGCCGGCACCGTCGACACTGGAAACAGTCACCTGC
Forward Primer for V_HH pDUAL-V_HH+linker+mNeon plasmid having linker in the middle vector used pDUAL-pef1a	CAGGTGACTGTTTCCAGTGTCGACGGTGCCGGCGCAGGAGCGatggtga
Forward Primer 5' UTR *mlt2*	ccctcgaggtcgacggtatcgataagcttTGGTGTCTTGAAACATTCGC
Reverse Primer for 5'UTR *mlt*1	gagggtattctgggcctccatgtcTTTCTCTTGCGTGCGAATTCCCTTA
Forward Primer for Hygromycin resistant *mlt2*	AAGGGAATTCGCACGCAAGAGAAAgacatggaggcccagaataccctcc
Reverse Primer for Hygromycin resistant *mlt2*	GCAACAAGTGTTTAAGCGCATACTcagtatagcgaccagcattcacata
Forward Primer for 3'UTR *mlt2*	atgtgaatgctggtcgctatactgAGTATGCGCTTAAACACTTGTTGCG
Reverse Primer for 3'UTR *mlt2*	ggccgctctagaactagtggatccAAAGGAACggaaggaaaagaaaagg
Bluescript II conformation primer	tattacgccagctggcgaaaggggg
Bluescript II conformation Reverse primer	Aggctttacactttatgcttccggc
Forward Primer for 5'UTR *wsc*1	ccctcgaggtcgacggtatcgataagcttACTGCAGCTAAAGAAGTTCT
Reverse Primer for 5'UTR *wsc*1	gagggtattctgggcctccatgtcGGAAATTAAATCGTGCGTAAGTATA
Forward Primer for Hygromycin resistant *wsc*1	ATACTTACGCACGATTTAATTTCCgacatggaggcccagaataccctcc
Reverse Primer for Hygromycin resistant *wsc*1	aaagtaaaaaagaaaaatgttgTTcagtatagcgaccagcattcacata
Forward Primer for 3'UTR *wsc*1	atgtgaatgctggtcgctatactgAAcaacatttttctttttacttta
Reverse Primer for 3'UTR *wsc*1	gtggcggccgctctagaactagtggatccaatttatccATACATTGCAT
Forward Primer for 5'UTR *sty*1	Ccctcgaggtcgacggtatcgataagcttcacattcttttttttttcga
Reverse primer for 5'UTR *sty*1	ggtgagttcaggctttttacccatTTTTATTCAAACTGGTTACAAAAAG
Forward Primer for Hygromycin resistant *sty*1	TTTTTGTAACCAGTTTGAATAAAAatgggtaaaaagcctgaactcaccg
Reverse Primer for Hygromycin resistant *sty*1	TTTAAGGCTTTATCTACAACTTGTttattcctttgccctcggacgagtg
Forward Primer for 3'UTR *sty*1	actcgtccgagggcaaaggaataaACAAGTTGTAGATAAAGCCTTAAAA
Reverse Primer for 3'UTR *sty*1	gtggcggccgctctagaactagtggatccAACACTTGCtagatgaaatt
Forward Primer for 5'UTR *atf*1	gaggtcgacggtatcgataagcttGTTGTTAGTTTACAGGAAACGTAAA
Reverse Primer for 5'UTR *atf*1	ggtgagttcaggctttttacccataattgaagaatttatGCTTTAACAC
Forward Primer for Hygromycin resistant *atf*1	TGTTAAAGCataaattcttcaattatgggtaaaaagcctgaactcaccg
Reverse Primer for Hygromycin resistant *atf*1	AGACCTTTTCAGATCAAAAACAGTttattcctttgccctcggacgagtg
Forward Primer for 3'UTR *atf*1	actcgtccgagggcaaaggaataaACTGTTTTTGATCTGAAAAGGTCTC
Reverse Primer for 3'UTR *atf*1	gtggcggccgctctagaactagtggatccTTTGTATACTAAAGCTTCGT
Forward Primer for 5'UTR *rgf1*	cccccctcgaggtcgacggtatcgataagcttACTTATAGCTTCTCATCC
Reverse Primer for 5'UTR *rgf1*	GGACGAGGCAAGCTAAACTGCTACTATAAAAACAATCAGTTTGACGATCG
Forward Primer for Kanamycin resistant *rgf*1	TGATTGTTTTTATAGTAGCAGTTTAGCTTGCCTCGTCCCC
Reverse Primer for Kanamycin resistant *rgf*1	TAAGTTCAAAAAAACCAAAAAAACTGGATGGCGGCG
Forward Primer for 3'UTR *rgf*1	CGCCATCCAGTTTTTTTGGTTTTTTTGAACTTAATTTTCACGTTTTATTT

Reverse Primer for 3'UTR *rgf* 1	cggccgctctagaactagtggatccTTCAAAAACATATCCTCTTTACCAG
Forward Primer for *rgf*1 His6	cccccctcgaggtcgacggtatcgataagcttCCACCAAACCCTCGTTTT
Reverse Primer for *rgf*1 His6	ggatccATAGGCGTGATGGTGATGGTGATGCATggatccTTGTTAACGTATATATTTTAAA

List of Oligonucleotides used in study for qPCR analysis

Supplementary Table S3

Primers	Sequence	Amplicon size in bp	Purpose
rgf1	ACGCCGACGTTTGTTAATTC<br>GAGCGTCCGACAAGTAAAGC		Expression check for the particular gene
rgf3	CCATTACCTGAAACGCCTGT<br>CCGGTGATCGAGTAGGTGAT		Expression check for the particular gene
gef2	TTGGCGAGAGTCAGTCATTG<br>AGGGTCTTCCCACCAAGAGT		Expression check for the particular gene
pck1	ATTAATATGCCAGCGGATGC<br>TCTCGGGGATCGTTGATAAG		Expression check for the particular gene

Supplementary Table S4: Table showing the predicted interaction between Rgf1 and V_HH (clone-C) protein.

Acceptor (Rgf1)	Donor (V_HH clone-C)	Hydrogen	Donor (V_HH clone-C) -Acceptor (Rgf1) Distance	Donor (VHH P-36)-Hydrogen-Acceptor (Rgf1) Distance
SER 149.A N	GLN 4.B OE1	SER 149.A HN	3.165	2.414
GLN 4.B NE2	SER 147.A O	GLN 4.B HE22	3.011	2.152
ARG 46.B NH1	LEU 150.A O	ARG 46.B HH11	3.517	2.614
SER 53.B OG	ASP 158.A O	SER 53.B HG	3.088	2.401
ARG 55.B NH1	ASP 158.A OD1	ARG 55.B HH12	2.675	1.768
ARG 55.B NH2	ASP 158.A OD1	ARG 55.B HH22	2.657	1.742
ARG 56.B NH1	PRO 164.A O	ARG 56.B HH11	2.921	1.959

Supplementary Video’s details

Supplementary Video-S1 DIC Wild Type- on YES DIC
Supplementary Video-S2 GFP Wild Type on YES GFP
Supplementary Video-S3 P36 in Wild Type EMM (-ura) DIC
Supplementary Video-S4 P36 in Wild Type EMM (-ura) GFP
Supplementary Video-S5 mtl2 on YES with P36 DIC-1
Supplementary Video-S6 mtl2 on YES with P36 GFP-1
Supplementary Video-S7 P36 in mtl2 EMM (-ura) DIC
Supplementary Video-S8 P36 in mtl2 EMM (-ura) GFP
Supplementary Video-S9 mtl2+P36_treatedSorbitol 1hr in YES
Supplementary Video-S10 mtl2+P36_treated Sorbitol 1hr in EMM (-ura)
Supplementary Video-S11 sorbitol wash off mlt2 on DIC YES medium
Supplementary Video-S12 sorbitol wash off mtl2 on GFP YES medium
Supplementary Video-S13 sorbitol wash off mtl2 on DIC EMM (-ura)
Supplementary Video-S14 sorbitol wash off mtl2 on GFP EMM (-ura)
Supplementary Video-S15 mtl2+P36_treated with KCl for 1hr in YES medium DIC
Supplementary Video-S16 mtl2+P36_treated with KCl for 1hr in YES medium
Supplementary Video-S17 KCl wash off mlt2 on_2 DIC YES medium
Supplementary Video-S18 KCl wash off mlt2 on_2 GFP YES medium
Supplementary Video-S19 KCl wash off mlt2 on_2 DIC YES medium
Supplementary Video-S20 KCl wash off mlt2 on_2 GFP YES medium
Supplementary Video-S21 KCl wash off mlt2 on DIC
Supplementary Video-S22 KCl wash off mlt2 on GFP
Supplementary Video-S23 H_2O_2 wash off mlt2 on YES DIC
Supplementary Video-S24 H_2O_2 wash off mlt2 on YES GFP
Supplementary Video-S25 H_2O_2 wash off mlt2 on YES DIC
Supplementary Video-S26 H_2O_2 wash off mlt2 on YES GFP
Supplementary Video-S27 clone C_time lapse_1 DIC Caspofungin
Supplementary Video-S28 clone C_time lapse_1 GFP Caspofungin
